# CAR-T cell therapy for glioblastoma: insight from mathematical modeling

**DOI:** 10.3389/fimmu.2025.1563829

**Published:** 2025-06-18

**Authors:** Magdalena Szafrańska-Łęczycka, Zuzanna Szymańska, Monika J. Piotrowska, Marek Bodnar, Urszula Foryś

**Affiliations:** ^1^ Doctoral School of Exact and Natural Sciences, University of Warsaw, Warsaw, Poland; ^2^ Interdisciplinary Centre for Mathematical and Computational Modelling, University of Warsaw, Warsaw, Poland; ^3^ Institute of Applied Mathematics and Mechanics, Faculty of Mathematics, Informatics and Mechanics, University of Warsaw, Warsaw, Poland

**Keywords:** CAR-T cell therapy, glioblastoma, cyclic treatment, single-dose strategies, mathematical modeling, numerical simulations

## Abstract

**Introduction:**

Glioblastoma is a rare, aggressive brain tumor marked by high therapeutic resistance, poor prognosis, and limited treatment options. Emerging immunotherapies, particularly Chimeric Antigen Receptor (CAR) T-cell therapy, offer promising alternatives to standard care. However, adapting CAR-T cell strategies from hematologic malignancies to solid tumors like glioblastoma presents substantial challenges.

**Methods:**

We extended existing mathematical models to investigate glioblastoma treatment dynamics with CAR-T cell therapy. Simulations were based on clinical trial-inspired scenarios targeting IL13Rα2, HER2, and EGFRvIII antigens, assessing both single-dose and cyclic dosing regimens. The models incorporated key biological processes, including tumor growth, CAR-T cell proliferation delays, and resistance mechanisms.

**Results:**

Cyclic CAR-T cell administration outperformed single-dose strategies in reducing tumor burden. Incorporating resistance and treatment delays into the models provided critical insights into relapse dynamics and therapeutic durability.

**Discussion:**

This study presents a comprehensive modeling framework for CAR-T cell therapy in glioblastoma, highlighting the importance of dosing regimens and resistance dynamics. The findings offer valuable guidance for optimizing therapeutic strategies to enhance patient outcomes.

## 1 Introduction

Glioblastoma is a rare, highly aggressive brain tumor characterized by therapeutic resistance and poor survival outcomes in both adults and children. Despite advancements in brain cancer research, translating insights into glioblastoma pathogenesis into improved clinical outcomes remains a critical unmet need Wu et al. (1) Dewdney et al. (2). The standard treatment for glioblastoma includes surgical resection followed by chemoradiotherapy Wu et al. (1) Migliorini et al. (3). However, robust DNA repair mechanisms and the self-renewing capacity of glioblastoma cells drive resistance to existing therapies, resulting in a 5-year survival rate of only 7.2% Wu et al. (1). Achieving durable glioblastoma management requires novel strategies, with research focusing on targeted molecular inhibitors, nanoparticle delivery systems, and immunotherapies Wu et al. (1).

Among immunotherapies, cellular approaches such as chimeric antigen receptor (CAR)-T cell therapy hold significant promise for treating brain tumors Dewdney et al. (2). Research on CAR-T cells began in the late 20th century Eshhar et al. (4), focusing on harnessing the immune system to combat cancer. In this therapy, a patient’s T cells are collected via apheresis and genetically modified *ex vivo* to express a chimeric antigen receptor (CAR) Dewdney et al. (2). The chimeric antigen receptor enables T cells to specifically recognize and eliminate tumor cells. After genetic engineering, the T cells are multiplied and reintroduced into the patient. In a clinical trial by Wrona and Potemski (5), 50.5% of patients with hematologic cancers survived at least two years after CAR-T therapy. This approach has also shown efficacy against colorectal cancer, breast cancer, and pancreatic ductal adenocarcinoma Kufel and Lewandowski (6). Current studies report BCMA-specific CAR-T cells achieving therapeutic responses in 20–64% of patients with relapsed or refractory multiple myeloma Cohen et al. (7). CAR-T therapy has recently been employed to treat B-cell acute lymphoblastic leukemia Pasvolsky et al. (8), with CD19-targeted CAR-T cells inducing complete remission in nearly 90% of patients with relapsed or refractory cases – significantly outperforming the less than 45% remission rate of conventional chemotherapy Park et al. (9).

Building on the success of CAR-T cell therapies in treating cancers, especially hematological ones, researchers are now working to extend these advances to aggressive solid tumors like glioblastoma. To aid this effort, mathematical models are being developed to better understand disease dynamics and treatment strategies. León-Triana et al. (10) proposed a four-dimensional mathematical model using differential equations to describe CAR-T cell therapy for glioma. The model incorporates two CAR-T cell populations (inside and outside the tumor), tumor cells, and B cells in dual-target treatment. It assumes exponential tumor growth and simulates a single CAR-T cell dose at treatment onset. Results suggest that single-target CAR-T cells require unrealistically high doses to overcome immune suppression, while dual-target CAR-T cells enable effective expansion and sustained tumor control. This approach holds promise for glioblastoma treatment, particularly when applied immediately after surgical resection and before cytotoxic therapy.

The model by León-Triana et al. (10) was later modified to explore CAR-T cell–tumor interactions under different treatment protocols. Bodnar et al. (11) introduced a two dimensional model focusing on CAR-T and tumor cell dynamics. While assuming exponential tumor growth, they analyzed multiple treatment scenarios, including continuous and intermittent CAR-T cell administration. Their results suggest that cyclic or continuous dosing is more effective than a single-dose strategy. A similar conclusion was reached in Bodnar et al. (12), which examined a modified four-dimensional model based on León-Triana et al. (10). This study incorporated logistic tumor growth and varied treatment regimens, highlighting that administering the highest possible dose at regular intervals outperforms a continuous moderate dose. Furthermore, intermittent dosing effectively extends tumor control, even with a lower average dose. Recently, Szafrańska-Łęczycka et al. (13) proposed a two-variable model, similar to Bodnar et al. (11, 12), but with a time delay to account for mitosis duration in cancer cell proliferation. This study focused on single-dose administration, enabling rigorous analysis of its effects. Results showed that complete tumor elimination was unachievable regardless of dose size, raising concerns about the long-term efficacy of single-dose treatments.

### 1.1 Clinical CAR-T approaches to glioblastoma treatment

Migliorini et al. (3) reported the results of three first-in-human CAR-T cell trials targeting IL13R*α*2, Her2/CMV, and EGFRvIII. We refer to these CAR-T cell therapy scenarios as the first, second, and third approaches to treatment, terms that are used consistently throughout the paper. The target antigen in the first approach to treatment was the IL13R*α*2 receptor. In this method, CAR-T cells were initially administered directly into the resection cavity using a Rickham catheter and subsequently into the brain’s ventricle. These CAR-T cells featured a mutated IL-13 domain (IL13 E13Y) in the antigen-binding site. Although significant tumor regression was observed following intraventricular administration, recurrence occurred later due to the loss of the IL13R*α*2 antigen. The second approach to the treatment targeted the HER2 receptor. CAR-T cells in this method were engineered to include the FRP5 (anti-HER2 scFv) domain, CD28 (for co-stimulation), and CD3*ζ* (for signal activation). The cells were administered intravenously at intervals of 6–12 weeks, with a maximum of seven cycles. These CAR-T cells remained in the body for up to 12 months, albeit in low numbers. One patient demonstrated a significant radiological response, while five patients experienced disease stabilization for over 24 months. The third approach to treatment involved a single infusion of CAR-T cells engineered with a humanized scFv domain targeting EGFRvIII, along with 4-1BB (for co-stimulation) and CD3*ζ* (for signal activation). These cells exhibited peak activity between days 3 and 10 but were undetectable after 30 days. Notably, one patient achieved disease stabilization lasting over 18 months. Comparing these approaches, the second method appears to be the safest, with doses ranging from 1 × 10^6^ to 1 × 10^8^ cells*/*m^2^ of patient body Migliorini et al. (3). This treatment regimen aligns with findings from Scharovsky et al. (14), which suggest that cyclic drug administration at regular intervals – typically in small doses with minimal rest periods – is the most effective strategy for cancer treatment. Indeed, administering the maximum tolerated dose in conventional therapies necessitates rest periods between treatment cycles, which can allow cancer to regrow and promote the emergence of therapy-resistant clones.

### 1.2 Research goals

Building on previous mathematical models, we aim to provide deeper insights into disease dynamics under CAR-T cell treatment. To achieve this, we modify earlier mathematical modeling attempts to reflect the treatment regimens described by Migliorini et al. (3). In the first approach, this involves incorporating a mathematical description of emerging resistance to treatment. The second and third approaches to treatment involve introducing a delay to account for the time required to activate CAR-T cells at the tumor site. We further present a comparison of the three approaches, which not only facilitates the identification of the most effective treatment strategies but also deepens our understanding of the factors influencing relapse or patient recovery. This comprehensive analysis provides valuable insights that can guide the optimization of CAR-T cell therapy protocols and improve patient outcomes.

Therefore, in this paper, our first goal is to reflect three CAR-T therapy scenarios studied in Migliorini et al. (3) using two simple modifications of mechanistic models proposed and analyzed in León-Triana et al. (10)–Szafrańska-Łęczycka et al. (13). In line with the treatment approaches Migliorini et al. (3), we consider a single-dose treatment strategy and cyclic CAR-T administration. We analyze the significance of the resistance to the therapy as well as the immune system’s response time to the administered CAR-T cells in the considered treatment strategies. Furthermore, we evaluate the potential benefits of cyclic CAR-T cell administration compared to a single-injection approach, and investigate the dose of CAR-T cells and the distance between subsequent doses required to achieve the best treatment outcomes. We perform sensitivity analysis, to find the models parameters most influential to the model’s dynamics. Naturally, the effects of treatment may vary depending on tumor size, the method of administration, and the administered dose of CAR-T cells. In the worst-case scenario, the tumor may grow to its maximum size while the CAR-T cell population diminishes entirely. Basing on our models suggestions we propose treatment strategies allowing for elongation of time to progression.

## 2 Materials and methods

### 2.1 Models

Let us begin by considering a basic model, which is a modified version of the one proposed by León-Triana et al. (10). It consists of two differential equations describing the temporal dynamics of glioma cells T(t)) and CAR-T cells C(t)) . In the original model by LeónTriana et al. (10), the authors assumed unlimited tumor growth. Considering this an oversimplification, we instead model tumor growth as constrained by the environment’s carrying capacity, using a logistic growth term. Note that this type of growth has a simple mathematical form. Yet, it can be considered representative of the variety of growth functions found in the literature [cf. the discussion on the biological background in Bodnar and Foryś (15)]. Here, we adopt a specific form of growth due to the quantitative nature of our analysis. However, from a mathematical perspective, qualitative results can often be obtained by making general assumptions about the growth function, as in previous work Bodnar et al. (12). Recognizing the importance of calibrating biological models, we are pursuing research in this direction, while acknowledging that it remains a complex task – even for simplified models Szymańska et al. (16) Gwiazda et al. (17).

Additionally, CAR-T cells actively eliminate tumor cells, a process represented by a bi-linear interaction term. The dynamics of CAR-T cells also account for their proliferation rate, inactivation, and natural death. The model, partially analyzed in Bodnar et al. (11, 12) and then in Szafrańska-Łęczycka et al. (13), is described by the following system:


(1) $$\begin{array}{l} \frac{d}{dt}T\left(t\right)=\rho_{T}T\left(t\right)\left(1-\frac{T\left(t\right)}{K}\right)-\alpha_{T}C\left(t\right)T\left(t\right),\\ \frac{d}{dt}C\left(t\right)=\left(\frac{\rho_{C}T\left(t\right)}{g_{T}+T\left(t\right)}-\frac{\alpha_{C}T\left(t\right)}{g_{C}+C\left(t\right)}-\frac{1}{\tau_{C}}\right)C\left(t\right), \end{array}$$

where all parameters are assumed to be positive. The parameter *ρT* represents the tumor growth rate, defined as the difference between the proliferation rate and the natural cell death rate. The term $\rho_{T}T\left(1-\frac{T}{K}\right)$ describes the development of a tumor in conditions of lack of immune system activity. The assumed logistic growth implies that the tumor’s expansion is limited, with *K* representing the tumor’s carrying capacity, or in other words, the maximum possible tumor size. We assumed that CAR-T cells eliminate cancer cells with an efficiency described by the parameter *α_T_*. The CAR-T cell dynamics equation includes three key processes. The first term describes CAR-T cell proliferation, which is triggered by contact with tumor cells and occurs at a maximum rate *ρ_C_*. Note that this process is saturated, meaning that it is proportional to the tumor size only for small tumors, while for larger tumors it tends to the constant value *ρ_C_*. Half saturation level *g_T_* reflects the tumor size for which proliferation reaches a half of maximal value *ρ_C_*. The second term models CAR-T cell inactivation by tumor cells at a maximum rate *α_C_*, with a half-saturation level *g_C_*. This inactivation reflects mechanisms such as CAR-T cell dysfunction induced by the tumor microenvironment. Finally, the third term accounts for the natural death or inactivation of CAR-T cells, with their average lifespan denoted by *τ_C_*.

Note that in the model Equation 1, we included only the most essential aspects of the considered therapy to maintain analytical tractability and minimize the number of parameters. The model does not account for key factors such as drug resistance or delayed immune response, which are crucial for accurately modeling the clinical trials discussed in Migliorini et al. (3). Therefore, the model would need to be adjusted accordingly.

The first approach to glioma treatment with CAR-T cell administration involved direct delivery through a Rickham catheter into the brain’s ventricle as described by Migliorini et al. (3). The treatment regimen began with the administration of an initial dose followed by additional administrations of CAR-T cells, again delivered via a Rickham catheter, at weekly intervals with the break before the last cycle, for detail description of the protocol see Subsection 2.2.3.

This method, although very promising at first, was associated with the rapid development of drug resistance. The precise mechanisms of this drug resistance remain unknown, but more information is available regarding its dynamics, thus we modeled it by introducing a time-dependent variable (*R*(*t*)). Hence, to model the first approach to treatment, we included an additional equation in the basic model, given by Equation 1, describing the strength of drug resistance:


(2) $$\begin{array}{l} \frac{d}{dt}T\left(t\right)=\rho_{T}T\left(t\right)\left(1-\frac{T\left(t\right)}{K}\right)-\alpha_{T}\left(1-R\left(t\right)\right)C\left(t\right)T\left(t\right),\\ \frac{d}{dt}C\left(t\right)=\left(\frac{\rho_{C}\left(1-R\left(t\right)\right)T\left(t\right)}{g_{T}+T\left(t\right)}-\frac{\alpha_{C}T\left(t\right)}{g_{C}+C\left(t\right)}-\frac{1}{\tau_{C}}\right)C\left(t\right),\\ \frac{d}{dt}R\left(t\right)=\alpha_{R}C\left(t\right)\left(1-R\left(t\right)\right). \end{array}$$

Drug resistance is modeled as reducing *α_T_* and *ρ_C_*, indicating that greater resistance leads to lower treatment efficacy and weaker CAR-T cell proliferation. The factor 1−*R*(*t*), which scales *α_T_* and *ρ_C_* in the first and second equations, respectively, represents this diminishing efficacy. Resistance changes over time proportionally to the CAR-T cell population, with self-inhibition preventing unlimited growth. A similar approach to modeling drug resistance was previously employed in Elishmereni et al. (18)Foryś et al. (19).

We follow this idea and therefore maintain a simple, minimally parameterized model, as noted earlier.

In the second and third approaches to glioma treatment described by Migliorini et al. (3), involving cyclic and single intravenous administration of CAR-T cells, respectively, drug resistance is negligible and excluded from the model. However, since CAR-T cells were administered intravenously, they required time to cross the blood-brain barrier and migrate to the tumor site. To address this, we introduced a time delay, *τ* (in days), representing the duration needed for CAR-T cells to reach the tumor, activate, and proliferate after interacting with cancer cells. Thus, the increase in activated CAR-T cells depends on the earlier states of *T*(*t*) and *C*(*t*) at time *t* − *τ*, leading to the following model


(3) $$\begin{array}{l} \frac{d}{dt}T\left(t\right)=\rho_{T}T\left(t\right)\left(1-\frac{T\left(t\right)}{K}\right)-\alpha_{T}C\left(t\right)T\left(t\right),\\ \frac{d}{dt}C\left(t\right)=\rho_{C}\frac{T\left(t-\tau \right)C\left(t-\tau \right)}{g_{T}+T\left(t-\tau \right)}-\left(\frac{\alpha_{C}T\left(t\right)}{g_{C}+C\left(t\right)}+\frac{1}{\tau_{C}}\right)C\left(t\right). \end{array}$$

The model, in this form has been mathematically analyzed by Szafrańska-Łęczycka et al. (13). It is worth noting that introducing a delay into the model may result in oscillatory behavior of the solutions. While such oscillations can occasionally be observed in medical treatments, they are generally undesirable in therapy and they can interfere with the oscillations caused by the cyclic administration of CAR-T cells.

### 2.2 Numerical simulation setup

#### 2.2.1 Parameters

All model parameters and their reference values are defined as positive constants and are summarized in **Table 1**. Note that the parameter *α_R_* appears only in model Equation 2. Although the presented models include eight main parameters, however, for model Equation 3 the delay *τ* is also treated as an additional parameter.

**Table 1:** Parameters of models Equations 2 and 3 together with the initial conditions defined by Equation 4: reference values, ranges and corresponding references.

Name	Description	Ref. value	Range	Units	Reference
*ρ_T_* *K* *α_T_*	tumor growth rate maximum tumor size inactivation of CAR-T cells by tumor	0.01 2 × 10^12^ 2.5×10^−10^	± 20% ± 20% ± 20%	day^−1^ cell day^−1^cell^−1^	Stein et al. (20) estimated León-Triana et al. (10)
*ρ_C_* *g_T_*	CAR-T proliferation tumor half-saturation level	0.9 1 × 10^10^	± 20% ± 20%	day^−1^ cell	Stein et al. (20) Stein et al. (20)
*α_C_*	CAR-T inactivation rate	0.05	± 20%	day^−1^	Radunskaya et al. (21)
*g_C_*	CAR-T half-saturation level	2 × 10^9^	± 20%	cell	Radunskaya et al. (21)
*τ_C_*	active CAR-T cell mean lifetime in the tumor site	7	± 20%	day	Ghorashian et al. (22)
*α*_R_ *τ*	drug resistance factor mean time needed to trigger the production of CAR-T cells in the organism	8 × 10^−10^ 2	± 20% ± 20%	day^−1^ day	estimated Qi et al. (23)
*T*_0_	initial average number of tumor cells	1.5 × 10^10^	± 20%	cell	estimated
*C*_0_	initial average number of CAR-T cells	2 × 10^7^ (☆) 5×10^8^ (☆☆)	± 20%	cell	Migliorini et al. (3) Bodnar et al. (12)
*R*_0_	initial cancer cell resistance	0	—	—	estimated

(☆) value used to perform the sensitivity analysis for model Equation 2, (☆☆) value used to perform the sensitivity analysis for model Equation 3.

#### 2.2.2 Initial conditions

To run simulations for considered models the initial conditions need to be defined. In the case of Equation 1, this means specifying the values of *T*(0) and *C*(0), which we assume to be:


(4) $$T\left(0\right)=T_{0}>0\;\mathrm{and}\;C\left(0\right)=C_{0}>0.$$

In the case of Equation 2, the model is extended by an equation describing acquired drug resistance. This equation also requires an initial condition to be defined at the beginning of the simulation, leading to the following initial condition:


(5) $$T\left(0\right)=T_{0}>0\;\mathrm{and}\;C\left(0\right)=C_{0}>0\;\mathrm{and}\;R\left(0\right)=R_{0}=0.$$

For delay differential equations, such as Equation 3, the initial conditions must be defined as a function on the interval [−*τ*,0]. In the absence of CAR-T cells, we assume that tumor growth follows a logistic pattern. Consequently, the initial condition for *T*(*t*) is defined as:


(6) $$T\left(t\right)=\frac{K}{1-\left(1-\frac{K}{T_{0}}\right)e^{-\rho_{T}t}},\;\text{for}\;t\in \left[-\tau ,0\right].$$

Since the effects of CAR-T therapy commence at *t* = 0, we assume C(t)=0 for t∈[−τ,0) , with a dose *C*_0_ administered at *t* = 0. These assumptions lead us to the following initial condition for *C(t)*


(7) $$C\left(t\right)=\{\begin{array}{ll} 0, & \text{for}\;t\in \left[-\tau ,0\right),\\ C_{0}>0, & \text{for}\;t=0. \end{array}$$

#### 2.2.3 Treatment protocols

The third approach to treatment assumes a single dose of CAR-T cells administered at the beginning of treatment. Modeling this approach is relatively straightforward, as it requires setting an appropriate initial condition, where the initial value, *C*_0_, corresponds to the administered dose. The first and second approaches to treatment described by Migliorini et al. (3), involve multiple doses of CAR-T cells administered at predefined time intervals.

**Figure 1:**
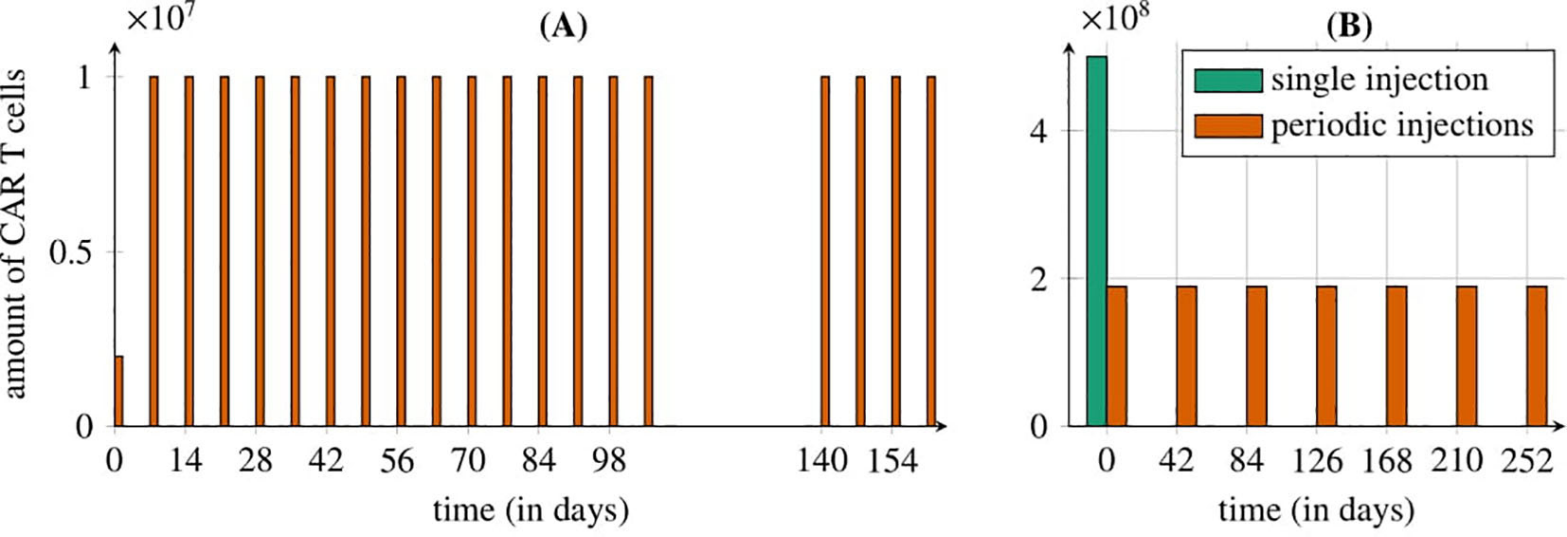
Representation of the treatment protocols studied by Migliorini et al. (3). **(A)** Cyclic injections of IL13R*α*2 CAR-T cells directly into the resection cavity via a Rickham catheter (first approach): starting with an intracerebroventricular dose of 2 × 10^6^ CAR-T cells, followed by 19 weekly cycles of 10×10^6^ cells, with a 5-week break between the 15th and 16th doses. **(B)** Intravenous administration of CAR-T cells in two ways (second and third approaches): seven cyclic HER2/CMV CAR-T injections every six weeks (1.89×10^6^ cells each, orange bars) or a single EGFRvIII CAR-T dose of 5×10^6^ cells (green bar). The treatment is modeled for a 70 kg, 180 cm patient with a tumor of 1.5 × 10^10^ cells.


These approaches necessitate a different model formulation to account for the repeated dosing schedule. We present them in concise form in **Table 2**. To mimic time-staggered dosing within the treatment regimen we used impulses to represent cyclically administered doses given at times *t_n_* = *nP*, where n∈ ℕ,n >1 , and *P* is the time interval between two consecutive cell administrations, see **Figure 1**. This leads to impulsive equations, where Equations 2 and 3 are solved for $t\in \left[t_{n},t_{n}+1\right)$ , with

**Table 2:** Summary of CAR-T cell treatment protocols targeting IL13R*α*2, Her2/CMV, and EGFRvIII.

Target	Administration route	Number of doses	Dose size	Model
IL13R*α*2	Intracranial (via Rickham catheter into the ventricle)	20	First dose: 2 × 10^6^ Subsequent doses: 1 × 10^7^	(2)
Her2/CMV	Intravenous	1–7	1.89 × 10^6^, 1.89 × 10^7^, or 1.89 × 10^8^	(3)
EGFRvIII	Intravenous	1	5 × 10^8^	(3)

$$T\left(t_{n}\right)=\underset{t\to t_{n}^{-}}{\lim}T\left(t\right),\;C\left(t_{n}\right)=\underset{t\to t_{n}^{-}}{\lim}C\left(t\right)+m,\;\left(t_{n}\right)=\underset{t\to t_{n}^{-}}{\lim}R\left(t\right)$$

for Equation 2, and

$T\left(t_{n}\right)=\underset{t\to t_{n}^{-}}{\lim}T\left(t\right),\;C\left(t_{n}\right)=\underset{t\to t_{n}^{-}}{\lim}C\left(t\right)+m$

for Equation 3, where *m* denotes a single CAR-T cell dose administered at time *t* = *nP*.

To evaluate the first approach targeting the IL13Rα2 receptor, we used the model defined by Equation 2 with initial condition (5). The first approach to glioma treatment with CAR-T cell administration involved direct delivery through a Rickham catheter into the brain’s ventricle as described by Migliorini et al. (3). The treatment regimen began with the administration of an initial dose of 2 × 10^6^ cells. This was followed by 5 doses of 1 × 10^7^ cells delivered via a Rickham catheter at weekly intervals, and an additional 10 doses of the same amount administered through a new Rickham catheter. After a 5-week break, the final 4 doses of 1 × 10^7^ cells were administered, also at weekly intervals. The schematic diagram of the treatment scheme is shown in **Figure 1A**, while the corresponding values are summarized in the first row of **Table 2**.

To evaluate the second approach targeting the HER2 receptor, we simulated the model described by Equation 3 with initial conditions (6) and (7). The simulations involved cyclic administration of seven equal CAR-T cell doses (an initial dose followed by six additional doses) at intervals ranging from 6 to 12 weeks, based on the methodology of Migliorini et al. (3). This methodology specifies that each CAR-T cell dose ranges from 1×10^6^ cells*/*m^2^ to 1 × 10^8^ cells*/*m^2^. We assume a sample patient with a body weight of 70kg and a height of 180cm. Using the Haycock formula, we calculated the range of doses that could be administered at each time point for the patient, see Haycock et al. (24). Consequently, each dose consider in our analysis falls within the range of 1.89×10^6^ to 1.89×10^8^ cells. The schematic diagram of the treatment scheme is shown in **Figure 1B**, while the corresponding values are summarized in the second row of **Table 2**.

To evaluate the third approach to treatment targeting EGFRvIII, we once again used Equation 3 with initial conditions (6) and (7), set to represent a single dose of CAR-T cells of 5 × 10^8^ cells administered at the start of the treatment. The schematic diagram of the treatment scheme is shown in **Figure 1B**, while the corresponding values are summarized in the third row of **Table 2**.

#### 2.2.4 Effectiveness of treatment protocols

To conduct a systematic study of the effect of dose distribution (dose size and time between injections) for the first approach to treatment we used model Equation 2 with initial condition (5). According to Migliorini et al. (3) we kept the first dose unchanged (2×10^6^ cells) while varied the size of subsequent doses and the time intervals between infusions, ensuring that the total administered amount of CAR-T cells maintain fixed (1.9 × 10^8^ cells) and equivalent to 19 doses of 10^7^ cells administered in the trail tested by Migliorini et al. (3).

A single dose size *d_i_* was calculated as:


(8) $$d_{i}=\frac{1.9\times 10^{8}}{i},\;\text{for}\;i=1,\ldots ,19.$$

For all tested protocols we compared tumor cell population 540 days after treatment initiation.

For the second approach to treatment, we utilized the model described by Equation 3 with initial conditions (6) and (7). We analyzed the significance of dose size, the number of subsequent doses, and the time interval between injections, following the methodology outlined by Migliorini et al. (3). Specifically, we examined equal-dose protocols throughout the entire treatment (including the initial doses), with single dose sizes of 1.89×10^6^, 1.89×10^7^, and 1.89×10^8^ cells, as detailed in Subsection 2.2.3. In other words, we focused on analyzing the effect of administering one to six additional doses, added to the initial dose, and the interval between them, on the treatment outcome. The impact of these treatment protocols on tumor cell population dynamics was evaluated 540 days after treatment initiation.

The efficacy of the third approach to treatment targeting EGFRvIII, modeled using Equation 3 with initial conditions (6) and (7), depended solely on a single dose of CAR-T cells administered at the start of treatment. Thus, it offered limited opportunities for manipulation or optimization.

To additionally evaluate the effectiveness of considered protocols we calculated time to progression (TTP) as it is a key measure for assessing treatment effectiveness in medicine. TTP is defined as the time (expressed in days) needed for the tumor to re-grow to the initial size i.e. to the size before the given treatment started.

#### 2.2.5 Tools

Numerical simulations and sensitivity analyses are conducted using MATLAB’s 4th-order Runge-Kutta method for Equation 2 and MATLAB’s 2nd-order Runge-Kutta method for Equation 3; see The MathWorks Inc. (25).

#### 2.2.6 Sensitivity analysis

Sensitivity analysis is a method used to evaluate how variations in a model’s parameters (called input) impact its dynamics (called output), see Saltelli et al. (26). We conducted sensitivity analyses of Equations 2 and 3 to identify the parameters that most significantly influence systems dynamics. Since we focused on the parameters of the mathematical models, in that part of the paper, we did not account for the cyclic administration of the drug in our analysis. Instead, we considered the case when the initial conditions reflect a single-dose administration of CAR-T cell therapy at the onset of treatment. In addition, we treated the delay value as an additional parameter for the system described by Equation 3. For both systems, we applied the Morris method as described in Qian and Mahdi (27), a widely used approach in biomedical modeling, particularly effective for nonlinear systems with potential parameter interactions. This method evaluates both the mean absolute effect, $\mu_{i}^{*}i=1,\ldots ,11$ ), which indicates the average impact of the *i*-th parameter (either a model parameter or an initial condition) on the model outcome, and the standard deviation of the effect, *σ_i_*, which reflects interactions or nonlinearities associated with the *i*-th parameter. The model outcome is defined as the average tumor cell count and CAR-T cell count over time, which serve as the basis for inferring two threshold quantities: the time at which the tumor cell population exceeds 3.5 × 10^10^ cells, and the time at which the CAR-T cell population drops below 1,000 cells. A high indicates a significant overall influence on the system, while a high *σ_i_* highlights parameters involved in complex interactions or nonlinear behavior, contributing to variability in outcomes. Accordingly, for both models, we plotted the rescaled values of and *σ_i_* for each considered model output. The rescaling process involved normalizing the data by the highest value(s) across all parameters for the analyzed measure to improve data presentation. The sensitivity analysis was performed using a uniform variation range of ±20% around the reference values for each parameter and initial condition, see **Table 1**.

Furthermore, in conducting the sensitivity analyses for the models, we focused on two specific (critical) thresholds: the point at which the cancer cell population grows to 3.5×10^10^ cells and the point at which the CAR-T cell population drops below 1000 cells. The justification for that choices is the following. Bogdańska et al. (28) indicated the typical astrocyte size to be approximately 10 *µ*m in diameter, with a cell density of 10^8^ cells mm^−3^. Thus, we estimated that a tumor with a radius of about 1 cm contains approximately 3.5 × 10^10^ cells. This value represents one-third of the lethal tumor burden, as determined by the radius measured using magnetic resonance imaging, according to Swanson et al. (29)Woodward et al. (30). A similar critical size threshold has also been proposed in Bodnar et al. (11, 12). On the other hand, monitoring the CAR-T cell population’s numbers, particularly when they fall below 1000, is crucial for predicting the patient’s long-term immune response to treatment. A low CAR-T cell count suggests that the body is unable to sustain an adequate immune response, which may adversely affect survival outcomes and contribute to disease progression. For a detailed description of the algorithm used, refer to the **Supplementary Material**.

## 3 Results

### 3.1 Numerical simulations of treatment protocols

The primary aim of developing the mathematical models was to numerically simulate the effects of glioblastoma treatment based on the therapeutic protocols outlined by Migliorini et al. (3). Considering the scope of the journal, the analytical work is provided in the **Supplementary Material**. The following section focuses on the visualization of our key results.

**Figure 2:**
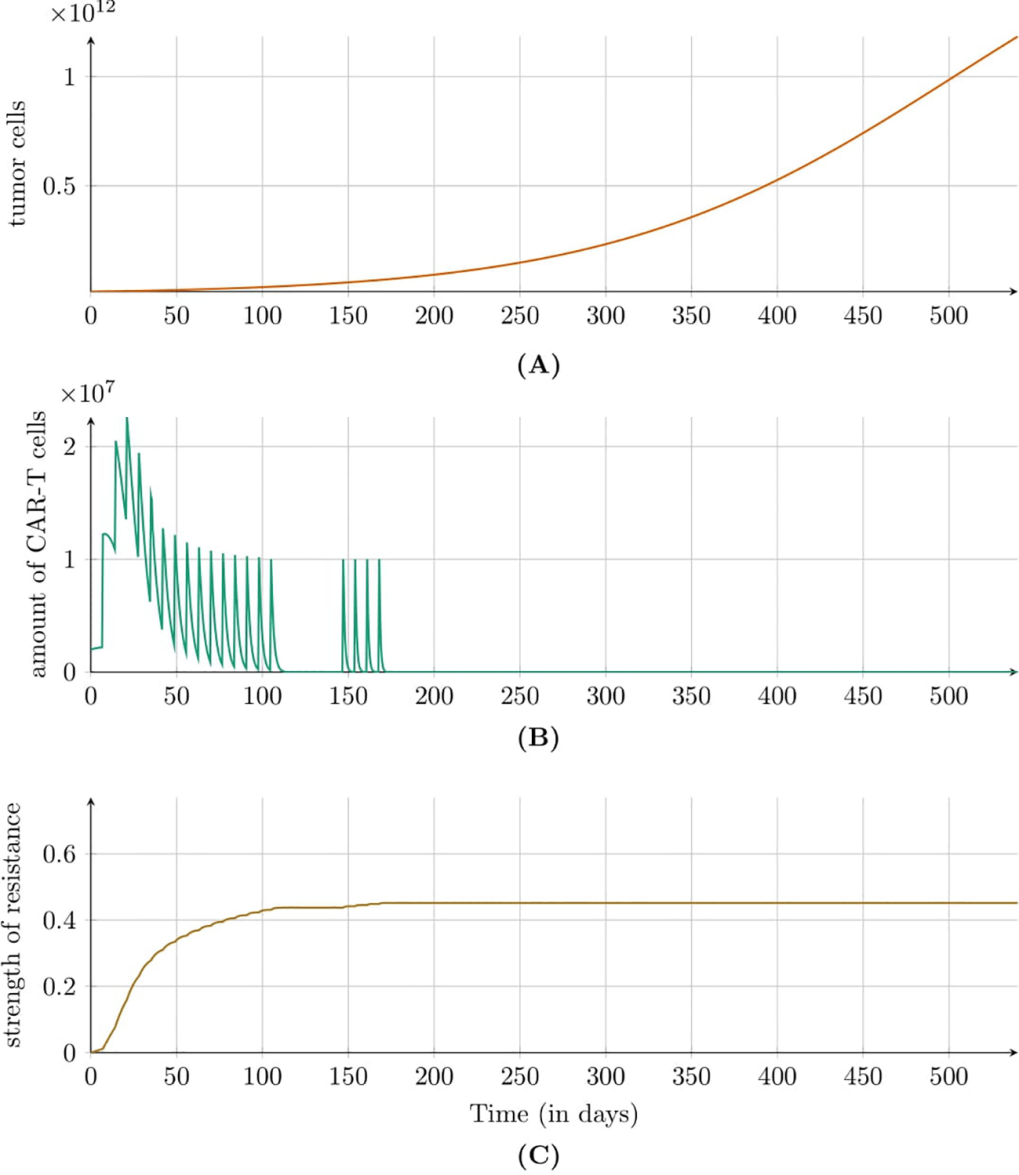
Dynamics of **(A)** tumor population, **(B)** CAR-T cell population, and **(C)** resistance strength modeled by Equation 2 with initial condition (5) under the first approach Migliorini et al. (3). Treatment included intracavitary CAR-T cell infusions via a Rickham catheter. Parameters and initial conditions are in **Table 1**, except *C*_0_ = 2 × 10^6^ CAR-T cells and the treatment schedule detailed in Subsection 2.2.3.

For the first approach to treatment targeting the IL13R*α*2 receptor (for details see Subsection 2.2.3) we see that despite the low level of drug resistance, the treatment had minimal impact, as the tumor, although growing slowly, continued to progress without showing any signs of regression, see **Figure 2**. Notably, an immune response was observed during the initial days of treatment, evidenced by an increase in CAR-T cell levels. Interestingly, the strength of drug resistance remained consistently below 0.5 throughout treatment.

**Figure 3:**
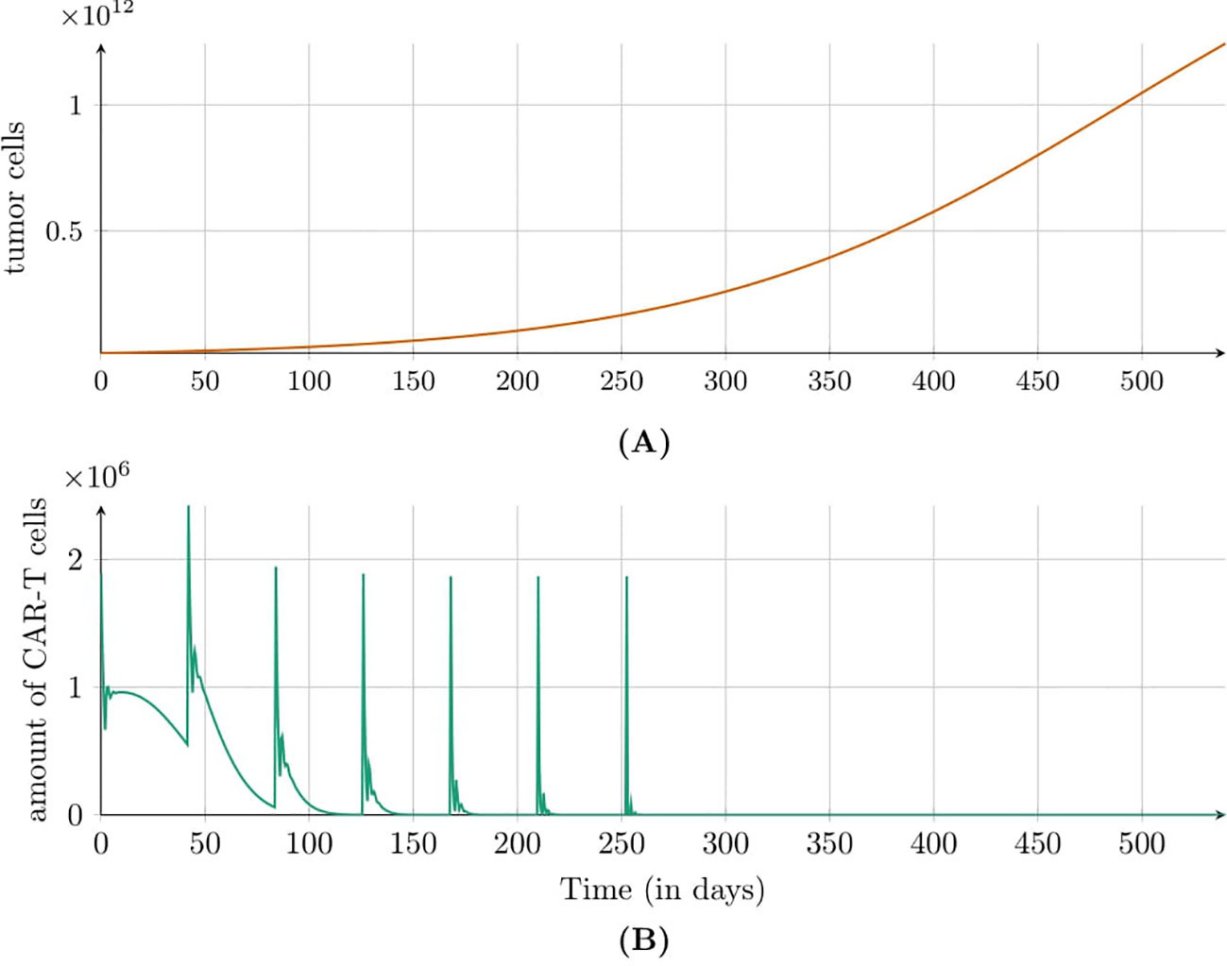
Dynamics of **(A)** tumor and **(B)** CAR-T cell populations modeled by Equation 3 with initial conditions (6) and (7) under the second approach Migliorini et al. (3). Treatment involved cyclic injections of 1.89 × 10^6^ HER2/CMV CAR-T cells every six weeks. Simulations assume a 70kg, 180cm patient. Parameters and initial conditions are in **Table 1**, except *C*_0_ = 1.89 × 10^6^ and the treatment schedule detailed in Subsection 2.2.3.

**Figure 4:**
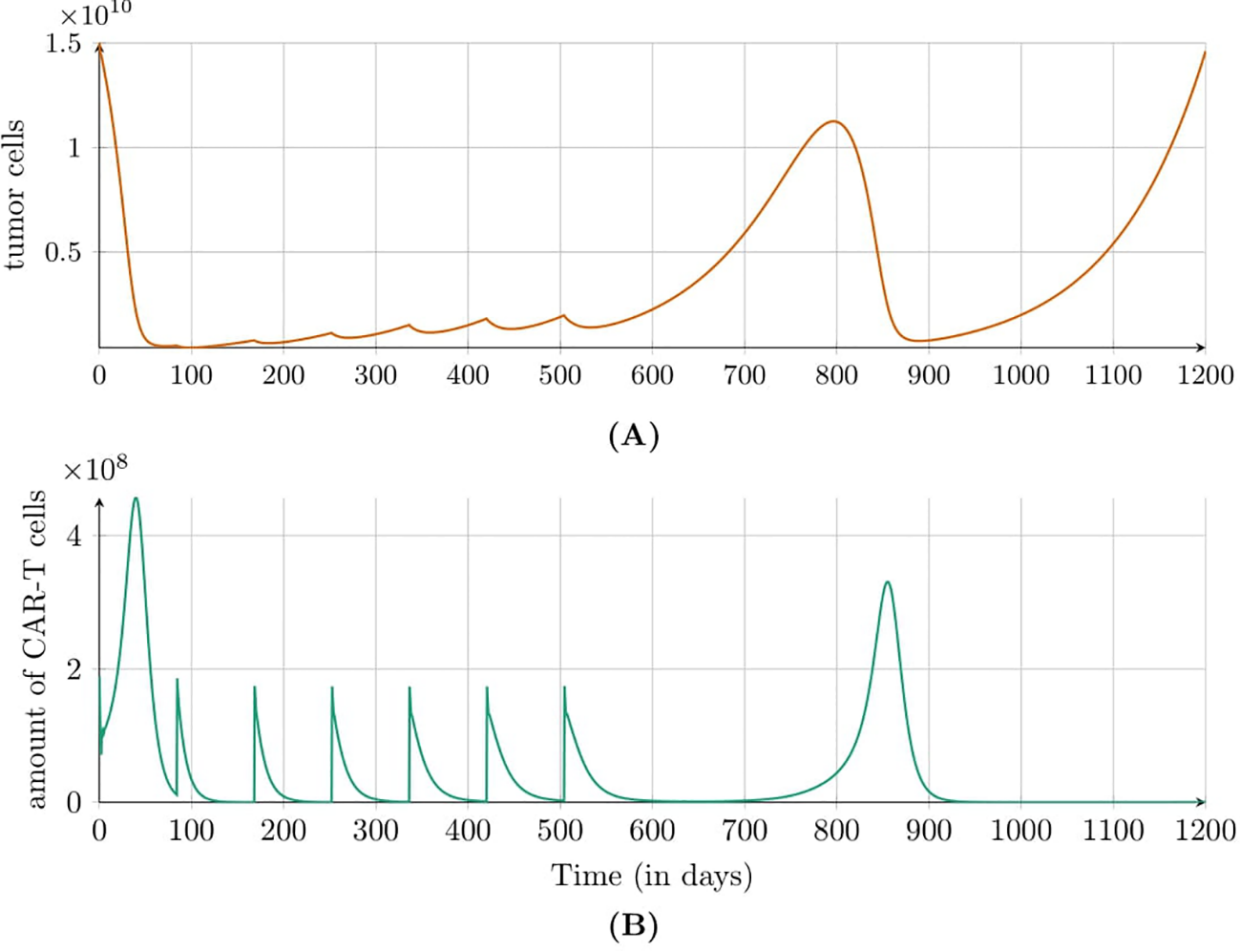
Dynamics of **(A)** tumor and **(B)** CAR-T cell populations modeled by Equation 3 with initial conditions (6) and (7) under the second approach Migliorini et al. (3). Treatment included cyclic injections of 1.89 × 10^8^ HER2/CMV CAR-T cells every 12 weeks. Simulations assume a patient weight of 70kg and height of 180cm. Parameters and initial conditions are listed in **Table 1**, except *C*_0_ set to 1.89 × 10^8^, and the treatment schedule detailed in Subsection 2.2.3.

**Figure 3** shows the simulation results for the second approach to treatment, which targeted the HER2 receptor involving a dose of 1.89 × 10^6^ CAR-T cells administered every six weeks. In this case, an immune system response was not observed, but the size of tumor increased steadily. **Figure 4** illustrates the results also for the second approach to treatment but this time with higher dose of 1.89 × 10^8^ CAR-T cells administered less frequently – every 12 weeks. A significant tumor reduction was observed, followed by a gradual regrowth that became more pronounced after the end of treatment. Interestingly, 300 days after treatment cessation, renewed tumor regression occurred due to a second pronounced immune system response. The time to progression (TTP) was extended to 1200 days.

**Figure 5:**
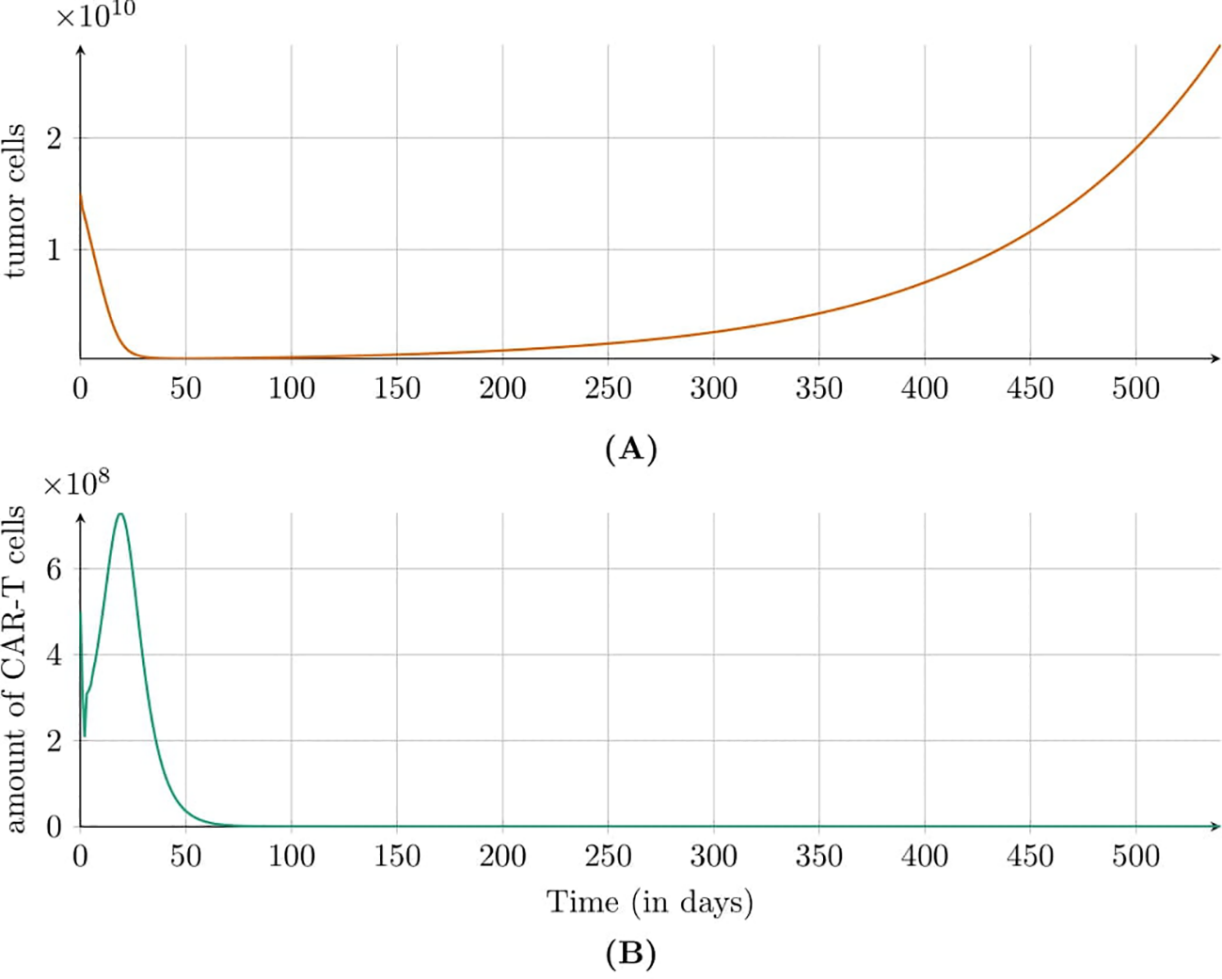
Dynamics of **(A)** tumor and **(B)** CAR-T cell populations modeled by Equations 3 with initial conditions (6) and (7) under the third approach Migliorini et al. (3). Treatment involved a single injection of 5 × 10^8^ EGFRvIII CAR-T cells. Parameters and initial conditions are listed in **Table 1**, with *C*_0_ marked by (☆☆) and the schedule detailed in Subsection 2.2.3.

Simulation results for the third approach to treatment targeting EGFRvIII receptor (for details see Subsection 2.2.3) indicated that CAR-T cells exhibited peak activity between days 3 and 10 but became undetectable by day 30, **Figure 5**, which is consistent with the behavior observed by Migliorini et al. (3). For a dose of 5 × 10^8^ cells, the TTP was approximately 425 days.

### 3.2 Results on treatment protocol effectiveness

An interesting and important issue is determining how the dose size and the time interval between individual administrations affect treatment effectiveness. For this reason, we investigated how the tumor cell population response depends on the CAR-T administration schedule, specifically varying dose sizes and intervals between injections according to the description in Subsection 2.2.4.

**Figure 6:**
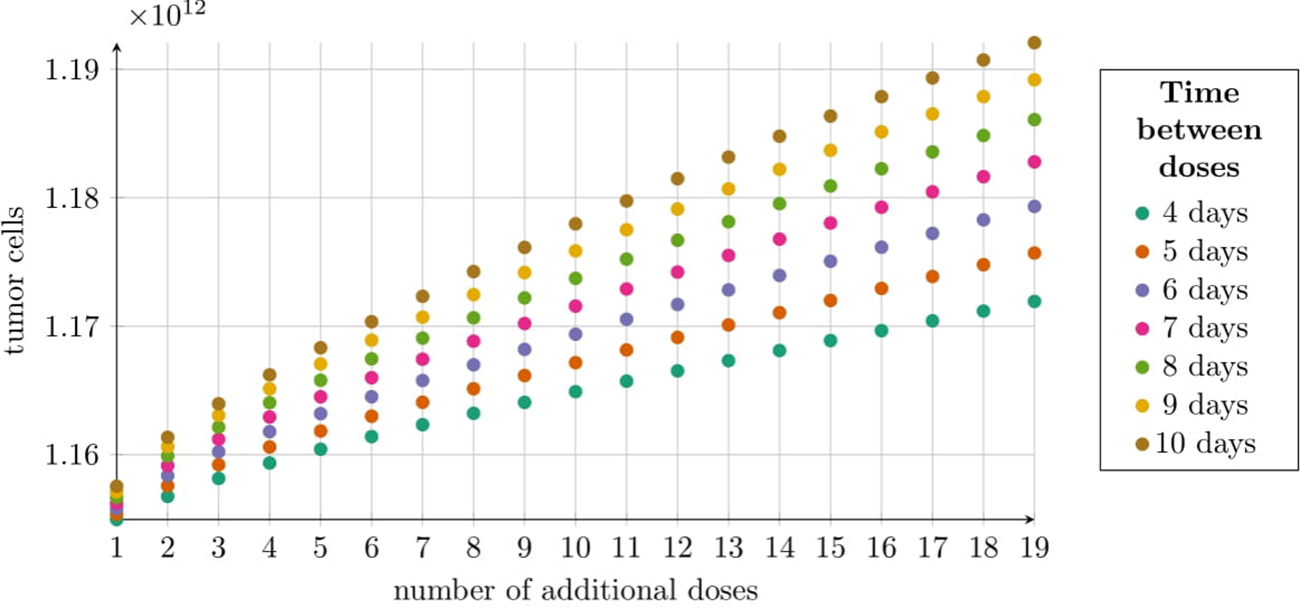
Tumor size at 540 days post-treatment under the first approach Migliorini et al. (3), modeled by Equation 2 with initial condition (5). Treatment began with 2 × 10^6^ CAR-T cells followed by 19 variable doses, totaling 1.9 × 10^8^ additional CAR-T cells (Subsection 2.2.4). The horizontal axis shows the number of CAR-T cell doses, with dosing intervals (4–10 days) indicated by colors in the legend. The vertical axis represents average tumor cell count at 540 days. Simulation parameters are in **Table 1**, except *C*_0_ = 2 × 10^6^ CAR-T cells. Cyclic doses were calculated using Equation 8.

Simulations of the first approach to treatment targeting the IL13R*α*2 receptor (see Subsection 2.2.3 and Subsection 2.2.4 for details) revealed that, with a fixed total number of CAR-T cells administered — equal to 1.9×10^8^ cells (note that this includes administrations in addition to the initial one of 2 × 10^6^ cells) — increasing the number of doses while decreasing the dose size reduced the treatment’s effectiveness. Shorter dosing intervals, however, resulted in a greater reduction in the tumor cell population after 540 simulation days, as shown in **Figure 6**.

**Figure 7:**
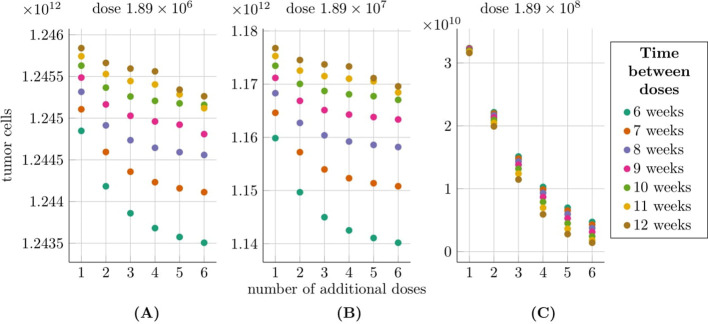
Tumor size at 540 days post-treatment under the second approach Migliorini et al. (3), modeled by Equation 3 with initial conditions (6) and (7), accounting for varying dose sizes and dosing intervals. The horizontal axis shows the number of additional CAR-T doses (excluding the initial dose), with colors indicating dosing intervals (6–12 weeks). The vertical axis represents the average tumor size 540 days after treatment initiation. Plots correspond to dose sizes: **(A)** 1.89 × 10^6^, **(B)** 1.89 × 10^7^, and **(C)** 1.89 × 10^8^ cells. Treatment was administered to a patient weighing 70 kg and measuring 180 cm, as described in Migliorini et al. (3). The simulation parameters are provided in **Table 1**, except for *C*_0_, which is the same as the considered dose size in each of the three scenarios.

Simulation results for the second approach to treatment targeting the HER2 receptor, as described in Subsection 2.2.4, are shown in **Figure 7**. The tumor cell population was analyzed 540 days after treatment initiation, considering varying CAR-T cell doses and dosing intervals. At the lowest dose [1.89×10^6^ cells, panel (A)], tumor cell levels remained largely unchanged, even with frequent dosing (every six weeks), fluctuating around 1.25 × 10^12^. The weakest effect occurred with a single dose administered 12 weeks after the initial dose. For the intermediate dose [1.89 × 10^7^ cells, panel (B)], slight tumor reductions were observed compared to the lowest dose. Increasing dosing frequency further reduced tumor cells, with six doses every six weeks lowering the population to approximately 1.14 × 10^12^. Less frequent dosing (every 12 weeks) maintained tumor levels between 1.17 × 10^12^ and 1.18 × 10^12^. At the highest dose [1.89 × 10^8^ cells, panel (C)], the most significant tumor reductions were seen. The best outcome, with six doses every 12 weeks, reduced tumor levels to below 1.41 × 10^9^. Notably, for the first two doses, higher dosing frequencies achieved better results, whereas at the highest dose, effectiveness improved with less frequent dosing.

**Figure 8:**
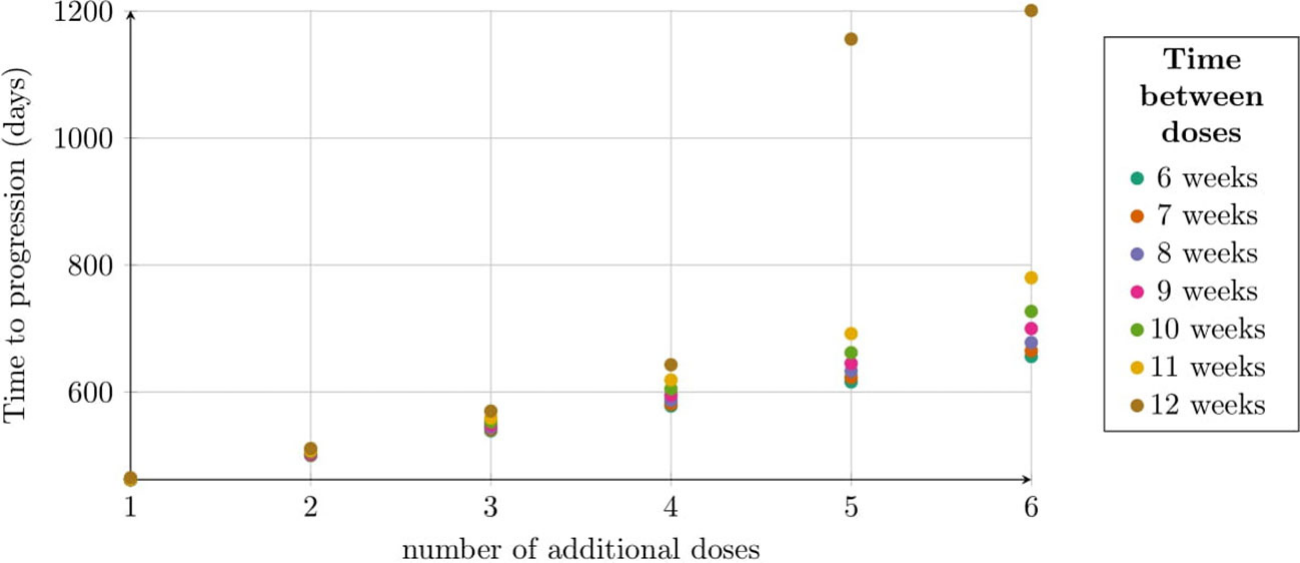
TTP distribution under the second approach, illustrating the impact of dose number and interval size. Each dose contained 1.89 × 10^8^ CAR-T cells. The horizontal axis shows the number of doses, with colors indicating dosing intervals (6–12 weeks). The vertical axis represents TTP (days). Simulation parameters are in **Table 1**, except *C*_0_ = 1.89 × 10^8^.

We also analyzed TTP for the second approach to treatment as a function of the number of doses and the intervals between them, as outlined in Subsection 2.2.4. Our analysis focuses on a dose of 1.89 × 10^8^, identified in previous simulations as the most effective. Notably, the longest TTP was observed with six doses administered every 12 weeks, while a slightly shorter TTP was achieved with five doses at the same interval, as shown in **Figure 8**. Interestingly, the addition of the fifth and sixth doses in the 12-week schedule significantly extended TTP by over 500 days. This improvement correlates with renewed tumor regression, driven by a second pronounced immune system response, which is shown in **Figure 4**.

**Figure 9:**
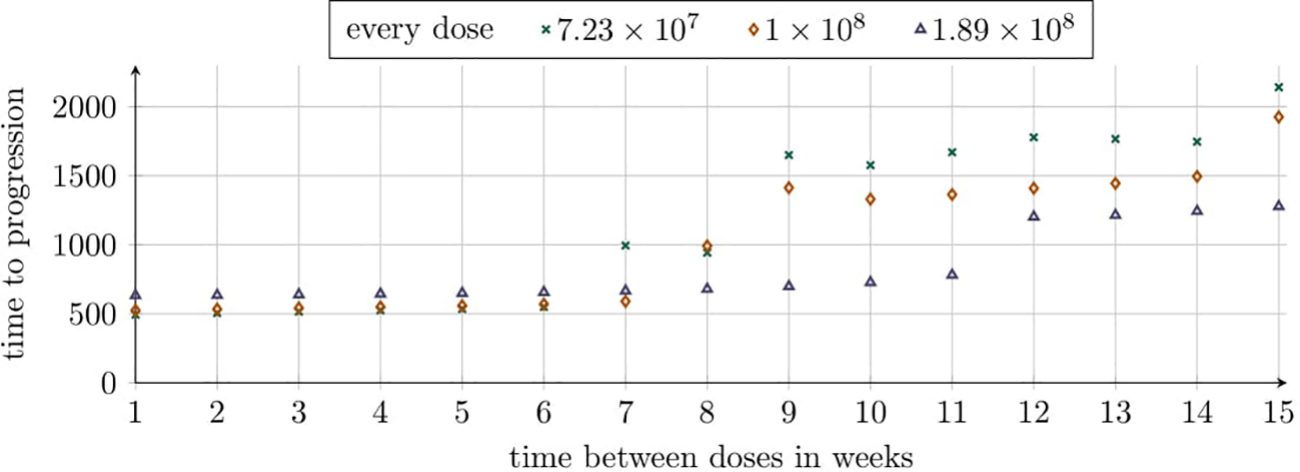
TTP distribution under the second approach, reflecting varying dosing intervals across simulations with constant intervals per simulation. Each simulation evaluates six additional doses of sizes 7.23×10^7^, 1×10^8^, and 1.89×10^8^. The horizontal axis represents dosing intervals (1–15 weeks), with markers indicating dose sizes as shown in the legend. The vertical axis represents TTP (days). Simulation parameters are detailed in **Table 1**, except *C*_0_ which matches the dose size for each scenario.

We evaluated the effectiveness of CAR-T cell treatment protocols involving seven equal doses – 7.23 × 10^7^, 1 × 10^8^, and 1.89 × 10^8^ cells – administered at intervals ranging from 1 to 15 weeks, as shown in **Figure 9**. The best outcome was achieved with the lowest dose (7.23 × 10^7^ cells) administered every 15 weeks, resulting in a TTP of 2140 days. For intervals of 1 to 6 weeks, the highest dose (1.89 × 10^8^ cells) was most effective, followed by the medium dose (1 × 10^8^ cells) and the lowest dose. However, with a 7-week interval, the ranking shifted, and the lowest dose provided the longest TTP of 994 days. At an 8-week interval, the medium dose (1 × 10^8^ cells) slightly outperformed the lowest dose, with TTPs of 992 and 942 days, respectively. For intervals of 9 to 15 weeks, the lowest dose consistently yielded the best outcomes, while the highest dose resulted in the shortest TTP.

**Figure 10:**
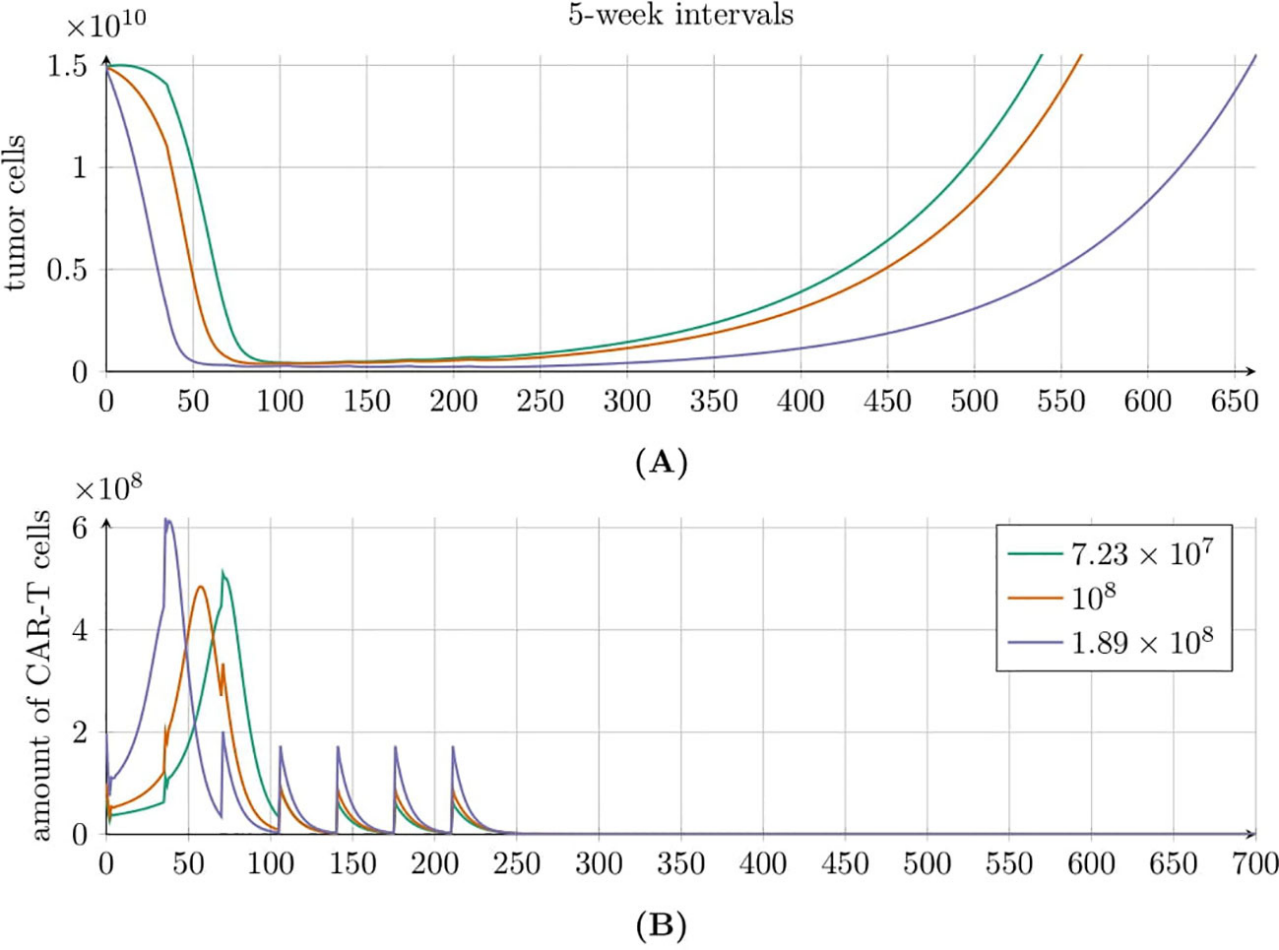
Tumor **(A)** and CAR-T cell **(B)** population dynamics modeled by Equation 3 with initial conditions (6) and (7), under the second approach Migliorini et al. (3). Seven equal doses of HER2/CMV CAR-T cells were administered at 5-week intervals, with individual dose sizes of 7.23 × 10^7^ (green line), 1 × 10^8^ (red line), and 1.89 × 10^8^ (purple line) cells. Parameters and initial conditions are provided in **Table 1**, except for *C*_0_ which matches the dose size for each of the three scenarios.

To understand the seemingly counterintuitive simulation result – where lower doses outperform higher doses at longer intervals – we performed additional time-solution simulations for the specified treatment protocols with intervals of 5, 10, and 15 weeks. Results for the 15-week intervals are provided in the *Supplementary Material*, see **Supplementary Figure S5**. In **Figure 10**, we examined the administration of seven doses of CAR-T cells with sizes 7.23×10^7^, 1×10^8^, and 1.89×10^8^ at 5-week intervals. The simulations revealed that each dose rapidly reduces the tumor cell population, followed by regrowth once CAR-T cells were no longer detectable in the body. The CAR-T cell population dynamics were similar across all dose sizes, likely due to the immunological response occurring at similar times. Notably, the highest dose (1.89 × 10^8^) most effectively delayed the tumor’s return to its original size.

**Figure 11:**
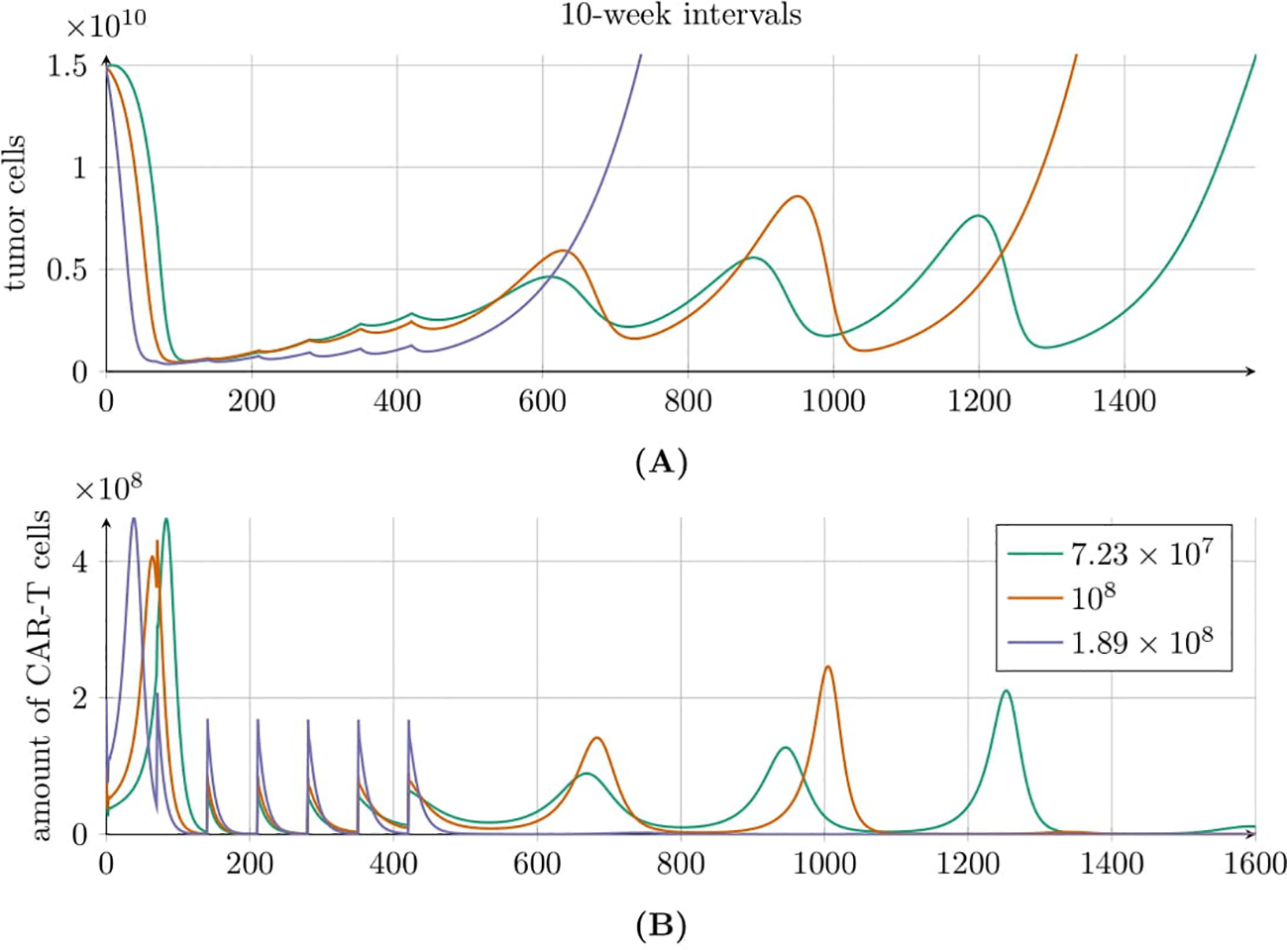
Tumor **(A)** and CAR-T cell **(B)** population dynamics modeled by Equation 3 with initial conditions (6) and (7), under the second approach Migliorini et al. (3). Seven equal doses of HER2/CMV CAR-T cells were administered at 10-week intervals, with individual dose sizes of 7.23 × 10^7^ (green line), 1 × 10^8^ (red line), and 1.89 × 10^8^ (purple line) cells. Simulations assume a 70kg, 180cm patient. Parameters and initial conditions are provided in **Table 1**, except for *C*_0_ which matches the dose size for each of the three scenarios.

A different behavior is observed in **Figure 11**, where seven doses of CAR-T cells were administered every 10 weeks. As before, each dose of CAR-T cells reduced the tumor cell population; however, the subsequent dynamics varied significantly. For the highest dose of 1.89 × 10^8^, we observed the fastest tumor recurrence. In contrast, the two lower doses resulted in oscillatory behaviors that ultimately lead to exponential tumor growth. Tumor regrowth to its original size occurred latest with the lowest dose of 7.23 × 10^7^. The CAR-T cell population also exhibited interesting dynamics. For the highest dose, the CAR-T cell population rapidly declined to zero. However, the two lower doses induced oscillatory dynamics, similar to the tumor population, resulting in consecutive immune responses to treatment. Notably, for the lowest dose, we observed the longest cumulative treatment effect, with the CAR-T cell population disappearing only after 1200 days.

### 3.3 Sensitivity analysis results

The sensitivity analysis for the tumor cell population *T* was conducted using the Morris method for two models: Equation 2 with initial conditions described by Equations 3 and Equation 5 with initial conditions described by Equations 6 and 7. Details of the methodology are provided in Subsection 2.2.6 and the *Supplementary Material*. Comprehensive results for the CAR-T cell population *C* are also available in the *Supplementary Material*. Sensitivity analysis was performed over the entire time course (for details, see the **Supplementary Material**). However, in the main text, we present results only for three representative times – 180, 360, and 540 days post-administration – and for two thresholds: the time for the tumor to reach 3.5 × 10^10^ cells, and the time until the CAR-T cell population drops below 1,000 cells.

#### 3.3.1 Sensitivity analysis results for the model with drug resistance

**Figure 12:**
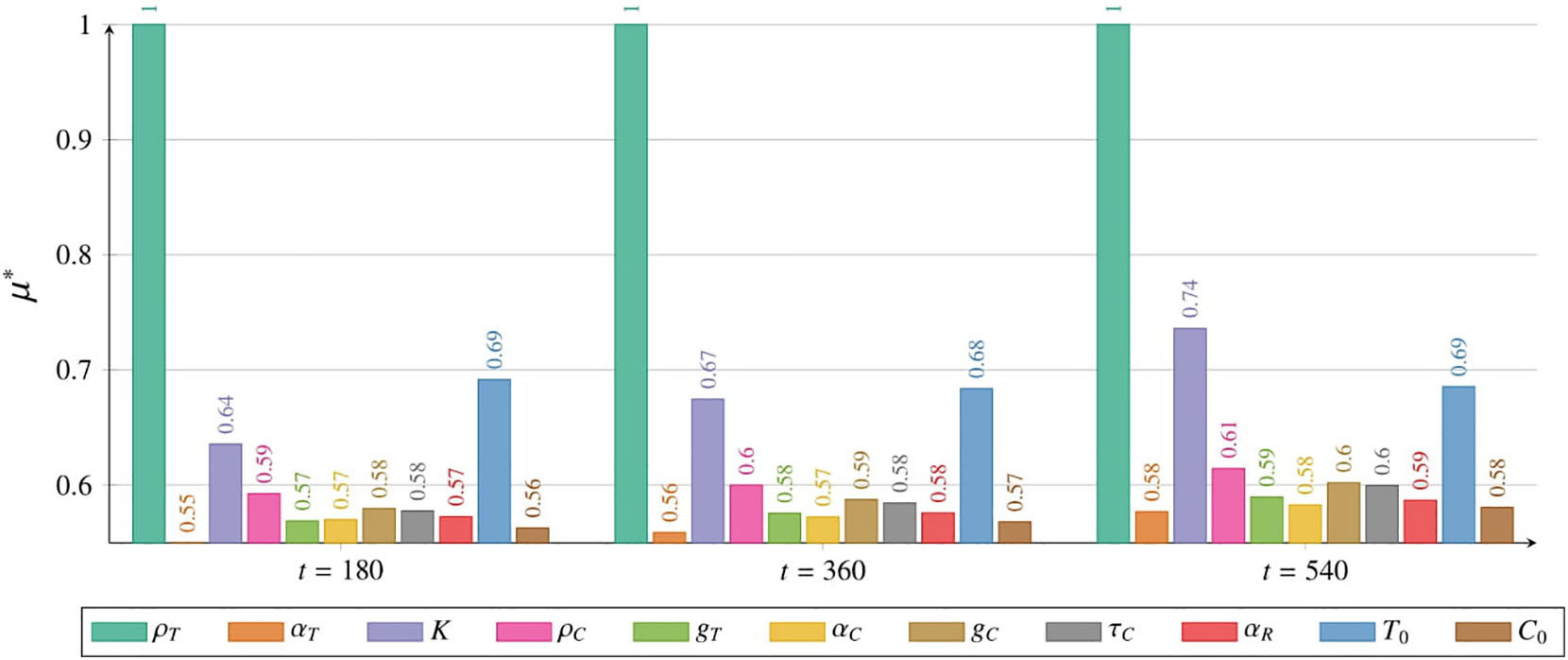
Impact of individual parameters and initial conditions on the tumor cell population, measured by *µ* ^∗^ (details in Subsection 2.2.6), as described by Equations 2 with initial condition (5), with a single CAR-T cell dose administered at *t* = 0. Results are shown for 180 days (left), 360 days (middle), and 540 days (right). Colors represent parameters or initial conditions as indicated in the legend. Simulation parameters are listed in **Table 1**, with *C*_0_ marked as (☆).

We analyzed the impact of individual parameters and initial conditions on the tumor cell population over time, based on Equation 2 with initial condition (5) (see **Supplementary Figure S6** in **Supplementary Material**). In **Figure 12** we presented results for three time points corresponding to 180, 360, and 540 days. Across all observed time points, the parameter *ρ_T_* had the greatest influence on the tumor cell population size. At 180 and 360 days, the second and third most influential factors were the initial tumor cell count *T*_0_ and the maximum tumor size *K*, respectively. However, after 540 days, the rankings of these two parameters had swapped. Other parameters had a significantly smaller impact on the tumor cell population size. Among these, the initial number of CAR-T cells *C*_0_ and the parameter *α_T_* describing the effective inactivation of CAR-T cells by the tumor had the smallest impact. In the *Supplementary Material* a comprehensive sensitivity analysis of both variables is presented, covering the period from day 0 (treatment initiation) to 540 days (1.5 years), see **Supplementary Figures S6** and **S7**.

**Figure 13:**
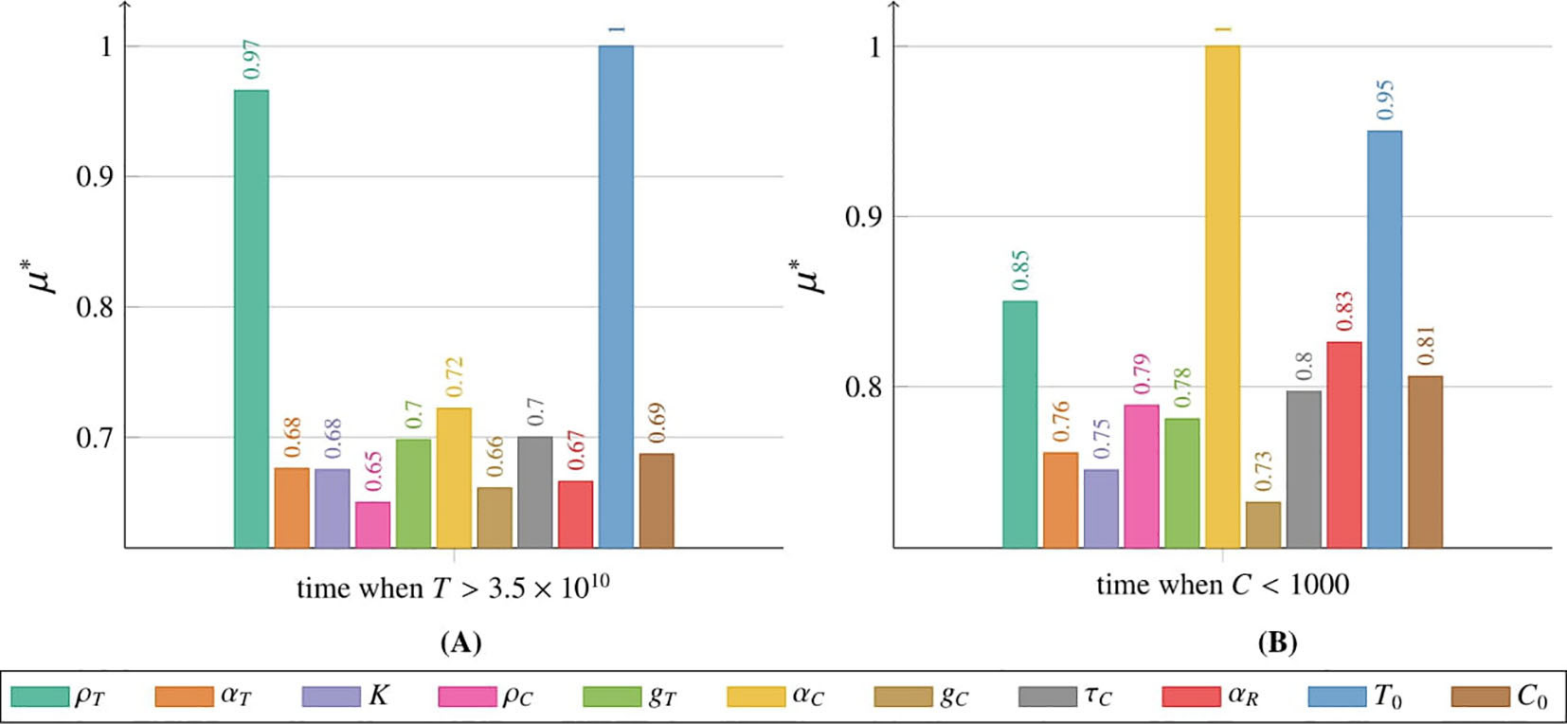
Impact of individual parameters and initial conditions on the tumor cell population, measured by *µ* ^∗^ (details in Subsection 2.2.6), as described by Equation 2 with initial condition (5), with a single CAR-T cell dose administered at *t* = 0. **(A)** Effect on *T* when the tumor population reaches 3.5 × 10^10^ cells, **(B)** effect on *C* when the CAR-T population drops below 1000 cells. Colors represent parameters and initial conditions, as per the legend. Simulation parameters are in **Table 1**, with *C*_0_ marked as (☆).

**Figure 13** illustrates the impact of parameter variations and initial conditions on two critical thresholds. Panel (A) depicts the effect of these variations on the time required for the tumor to reach the critical threshold of 3.5 × 10^10^ tumor cells, while panel (B) shows their effect on the time until the CAR-T cell population declined below the critical threshold of 1000 cells. Among the analyzed factors, the initial tumor size *T*_0_ was identified as the most influential determinant of the time to exceed the critical threshold for the tumor variable *T*. The tumor growth rate *ρ_T_* exhibited a nearly equal impact. In contrast, other parameters had considerably smaller effects, with *ρ_C_*, governing the proliferation rate of CAR-T cells, showing the least influence on the time required for the tumor to reach its critical threshold. Regarding the variable *C* and the time at which the CAR-T cell population falls below the critical threshold of 1000 cells, the most influential parameter was *α_C_*, representing the inactivation rate of CAR-T cells. Notably, the tumor growth rate *ρ_T_* and the initial tumor size *T*_0_, both critical for determining the time required for the tumor to reach its strategic threshold, also had significant effects in this context. This finding highlights the interconnected dynamics between the tumor and CAR-T cells. Another key parameter was *α_R_*, which represents the strength of drug resistance and underscores its crucial role in shaping treatment outcomes as modeled by Equation 2. In contrast, the CAR-T half-saturation level *g_C_* had the least influence.

#### 3.3.2 Sensitivity analysis results for the model with time delay

**Figure 14:**
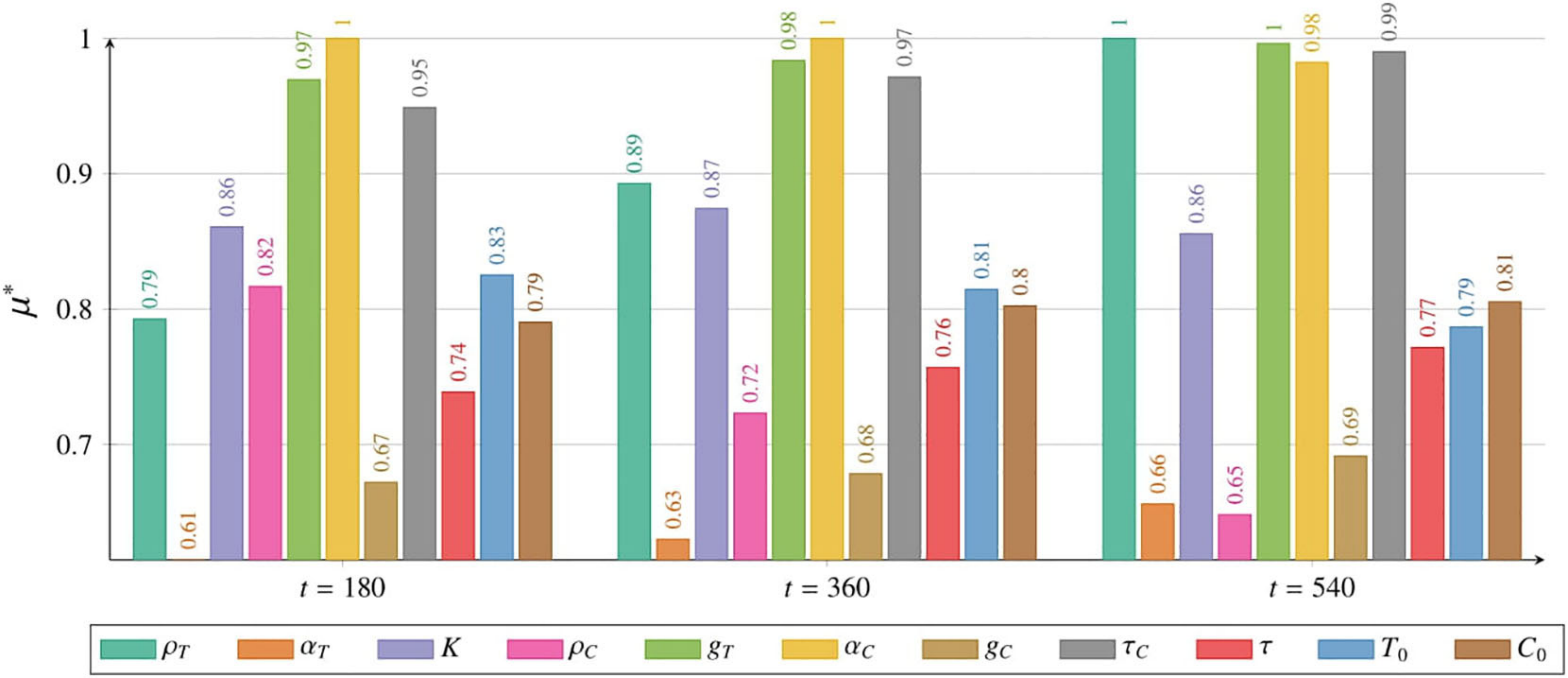
Impact of individual parameters and initial conditions on the tumor cell population, measured by *µ* ^∗^ (details in Subsection 2.2.6), as described by Equation 3 with initial conditions (6) and (7), with a single CAR-T cell dose administered at *t* = 0, as in the third approach Migliorini et al. (3). Results are shown for 180 days (left), 360 days (middle), and 540 days (right). Colors represent parameters or initial conditions, as indicated in the legend. Simulation parameters are listed in **Table 1**, with *C*_0_ marked as (☆☆).

Following the methodology presented in Subsection 2, we performed a series of simulations to study how changes in parameter values and initial conditions influence the tumor cell population. These simulations were based on Equation 3 with initial conditions specified in Equations 6 and 7, evaluated at three time points: 180, 360, and 540 days, see **Figure 14**. After 180 days, *α_C_*, representing the inactivation rate of CAR-T cells, exhibited the greatest influence on tumor population size. The next most influential parameters were the tumor half-saturation level *g_T_* and the mean lifetime of active CAR-T cells at the tumor site, *τ_C_*. The remaining parameters had a comparable impact on the dynamics of the tumor population, except for *α_T_*, which describes the effective inactivation of CAR-T cells by the tumor. This parameter had the least effect at this time point. Similar trends were observed after one year, with the tumor growth rate *ρ_T_* and *τ_C_* gaining importance. By 540 days, however, *ρ_T_* and *g_T_* emerged as the most critical determinants of tumor population dynamics. Notably, the parameters *α_C_* and *τ_C_* continued to play a significant role. An interesting observation at the last time point was the increased importance of the initial CAR-T cell value, *C*_0_, and the *α_T_* parameter. Additionally, *ρ_C_*, which governs the CAR-T cells proliferation, had the least impact on tumor population dynamics at this stage. The **Supplementary Material** include a detailed sensitivity analysis conducted from day 0 (the start of treatment) to day 540 (corresponding to 1.5 years) for both variables, see **Supplementary Figures S9** and **S10**. In addition to comprehensive plots for the measure, the materials also provide detailed plots for the *σ_i_* measure. For further details, see Subsection 2.2.6.

**Figure 15:**
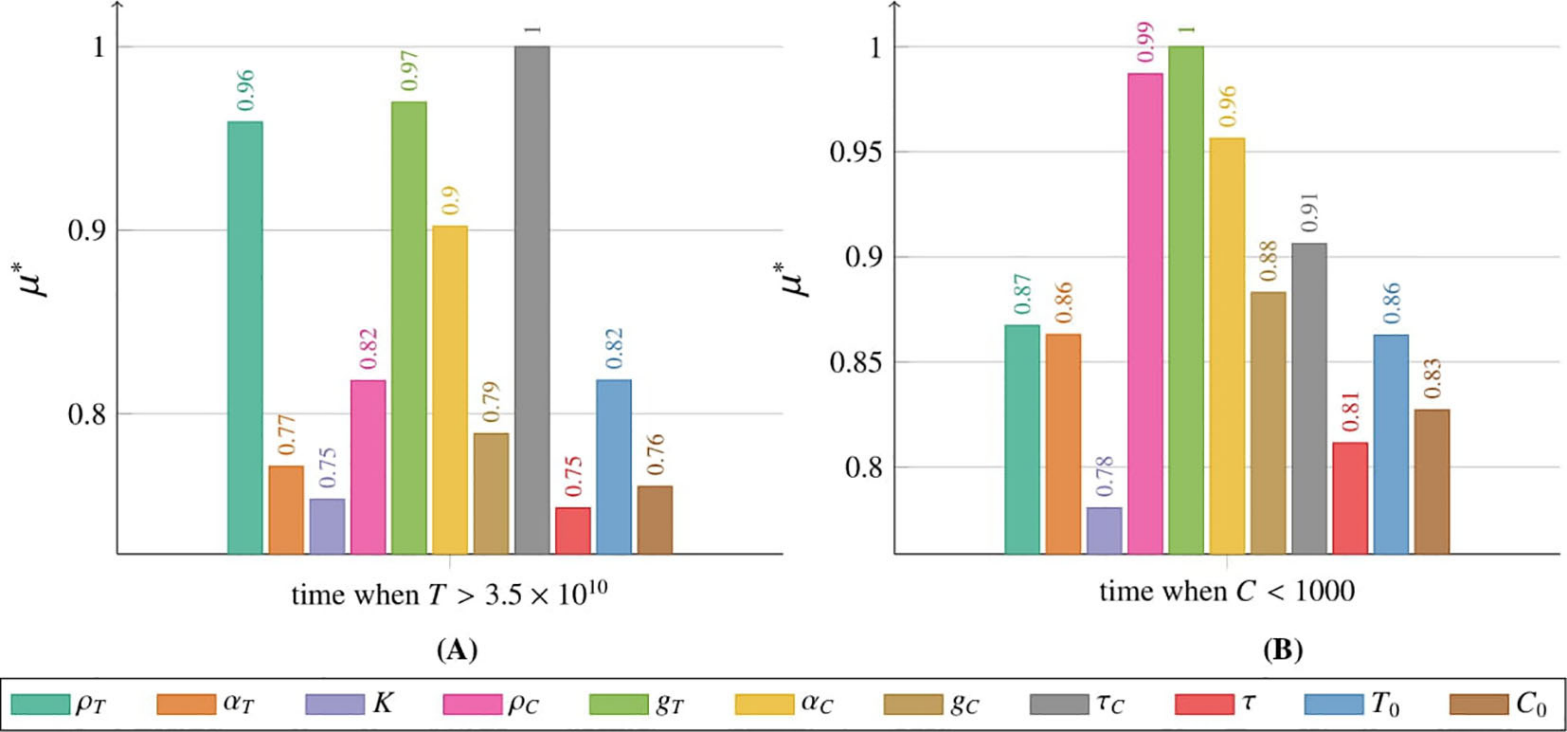
Impact of individual parameters and initial conditions on the tumor cell population, measured by *µ* ^∗^ (details in Subsection 2.2.6), as modeled by Equation 3 with initial conditions (6) and (7), with a single CAR-T cell dose administered at *t* = 0, as in the third approach Migliorini et al. (3). **(A)** Effect on *T* when the tumor reaches the critical threshold of 3.5×10^10^ cells, **(B)** effect on *C* when the CAR-T population falls below 1000 cells. Colors correspond to parameters and initial conditions, as indicated in the legend. Simulation parameters are listed in **Table 1**, with *C*_0_ marked as (☆☆).

We also investigated how variations in parameter values and initial conditions influence the time required for the tumor population to reach the critical threshold of 3.5 × 10^10^ cells (**Figure 15A**) and the time at which the CAR-T cell population falls below 1000 cells (**Figure 15B**). The parameter with the greatest influence on the time for variable *T* to reach the critical threshold of 3.5×10^10^ tumor cells was *τ_C_*, which represents the mean lifetime of active CAR-T cells at the tumor site. This was followed by *ρ_T_* and *g_T_*, which correspond to the tumor growth rate and the tumor half-saturation level, respectively. Interestingly, *τ*, representing the average time required to initiate CAR-T cell production in the body, and *K*, denoting the maximum tumor size, had the least impact on the tumor population threshold. Conversely, *g_T_* exerted the most significant influence on the time at which the CAR-T cell population fell below the critical threshold of 1000 cells. Notably, *ρ_C_*, which defines the CAR-T cell proliferation rate, exhibited a nearly comparable impact. In contrast, *K* was the least influential parameter in this context.

### 3.4 Supporting results

In the **Supplementary Material**, we provide additional analytical results related to models (2) and (3) under cyclic administration of CAR-T cells. These results are not included in the main text as they are not central to the primary focus of the study. We also present numerical illustrations demonstrating the impact of resistance (**Supplementary Figures S1**, **S2**) and time delay (**Supplementary Figures S3**, **S4**) on the model dynamics.

## 4 Discussion

Analyzing the simulation results for Equation 2 with initial condition (5), modeling the first approach to glioblastoma treatment (see Subsection 1.1), revealed a critical insight: the emergence of drug resistance effectively nullified the therapeutic effect. All tested dosing regimens produced similar outcomes within the assumed parameters range, as the rapid emergence of resistance rendered the therapy ineffective, see **Figure 2**. This finding aligns with clinical observations, where patients initially responded favorably to treatment but soon experienced diminishing therapeutic benefits. Therefore a deeper understanding of the resistance phenomenon is essential for accurately modeling disease dynamics and assessing treatment effectiveness. As an initial step, incorporating a delayed onset of drug resistance into the model could offer valuable insights. However, due to the lack of precise data on the timing of this delay, we opted to proceed with a simplified model that omits this feature. Exploring the delay duration and other characteristics of the resistance phenomenon is deferred to future research.

Regarding intravenous administration, as employed in the second and third approaches to treatment, clinical studies indicated that while the initial effects may be less pronounced, this method demonstrated greater long-term efficacy, particularly at higher and multiple doses. **Figures 3**, **4** illustrate simulations of treatment following the second approach, enabling an *in silico* comparison of therapeutic efficacy at low and high doses, respectively. Administering a single dose at the start of treatment, as in the third approach (see **Figure 5**), or insufficient dosing in the second approach failed to achieve significant tumor reduction. However, higher doses in the second approach yielded improved outcomes, especially when delivered through multiple administrations. This strategy resulted in a longer TTP compared to single-dose regimens, emphasizing the importance of optimizing dosing strategies, which can be effectively explored *in silico*.

### 4.1 Discussion on the effectiveness of treatment protocols

Treatment approaches based on the cyclic administration of CAR-T cells showed significant promise for improving therapeutic effectiveness. This was particularly true for the second approach, where the impact of resistance was less pronounced, although some conclusions can also be drawn for the first approach. Simulations highlighted the advantages of administering higher doses at shorter intervals, as illustrated in **Figure 6**. This effect was likely associated with the induction of resistance to treatment. For IL13R*α*2 receptor-targeted therapy, the optimal strategy may be to maximize tumor reduction with high doses of CAR-T cells while they remain effective, that is, before resistance develops.

In pursuit of guidelines for more effective therapeutic procedures, we focused particularly on the second approach to treatment. The analysis of simulations with varying dosages, as shown in **Figure 7**, yielded several notable conclusions. For the lowest dosage considered (1.89 × 10^6^ cells) and the moderate dosage (1.89 × 10^7^ cells), the optimal strategy was to administer CAR-T cells as frequently as possible. In contrast, the highest dosage (1.89×10^8^ cells) achieved the best results when administered less frequently, approximately every 12 weeks. This discrepancy likely arised because smaller doses may be insufficient to significantly inhibit tumor growth or effectively stimulate CAR-T cell proliferation. In such cases, frequent administration helped maintain a minimum therapeutic concentration, preventing excessive tumor regrowth between doses and gradually weakening the tumor cell population. Consequently, frequent administration of smaller doses became crucial for achieving any therapeutic effect. Conversely, higher doses allowed entry into the optimal therapeutic window, where a balance was achieved between tumor cell destruction and CART cell proliferation. This ensured maximum effectiveness, eliminating a substantial portion of the tumor while minimizing strain on the body and avoiding premature tumor saturation. Thus, higher doses administered at appropriately longer intervals yielded superior outcomes compared to frequent administration of high concentrations, which can reduce overall treatment efficacy.

While investigating the optimal therapeutic outcome for the highest dose considered, 1.89 × 10^8^ CAR-T cells, we identified several noteworthy conclusions regarding TTP. The most promising results were observed with five and six doses administered at 12-week intervals, yielding TTP values of 1156 and 1201 days, respectively. These values were significantly higher than those achieved with other administration schedules (see **Figure 8**). Such elevated TTP values were likely attributable to the secondary induction of an immune response – CAR-T cell activation triggered by the moderate regrowth of the tumor while sufficient CAR-T cells remained present to initiate the response. Regular administration of CAR-T cells at 12-week intervals appeared to optimize their therapeutic potential by balancing tumor cell elimination with CAR-T cell activation, effectively maintaining CART cells at a therapeutic level. An intriguing observation was that with a small number of additional doses – particularly a single additional dose – the TTP remained relatively consistent regardless of the dosing interval. These findings underscored the importance of both the number of doses and the dosing interval as critical parameters influencing the efficacy of CAR-T therapy. Repeated, regular administration of CAR-T cells at carefully selected intervals yielded the most favorable therapeutic outcomes.

Exploring the role of intervals between CAR-T cell administrations within the framework of the second approach to treatment revealed valuable insights. With fixed dose sizes (7.23×10^7^, 1×10^8^, and 1.89×10^8^ cells) administered over seven cycles, therapeutic efficacy highly depended on the time between administrations, see **Figure 9**. The most significant finding was that increasing the intervals between administrations and using a smaller dose led to better therapeutic outcomes. Specifically, the best result was achieved with the dose of 7.23 × 10^7^ cells (the smallest of the three doses considered) when administered every 15 weeks (the longest interval examined), resulting in a TTP of 2140 days.

To shed more light on the dynamics of tumor and CAR-T cell populations under the second approach to treatment and to better understand this counterintuitive result, we conducted additional numerical simulations of model Equation 3 with initial conditions specified in Equations 6 and 7, exploring varying dose sizes and administration intervals. **Figure 10** presents results for CAR-T cell administrations at 5-week intervals. In this scenario, lower CAR-T cell doses failed to reduce tumor cell levels sufficiently, resulting in suboptimal treatment outcomes, whereas higher doses yielded better results. In contrast, **Figure 11** shows results for 10-week intervals. Here, larger CAR-T doses caused a faster and deeper reduction in the tumor population and maintained it at low levels for longer. However, because CAR-T cell proliferation depended on the presence of tumor cells, excessive tumor suppression leaded to insufficient proliferation of new CAR-T cells and a rapid decline in their levels. Paradoxically, therefore, higher doses overly suppressed tumor cells, weakening the immune response and eventually depleting CAR-T cells. This loss of immune control allowed tumor cells to proliferate unchecked. Conversely, smaller doses maintained tumor cells at levels that are low enough to slow disease progression but high enough to sustain immune system activity, resulting in a longer TTP.

The results resemble those observed in the metronomic approach to chemotherapy, which suggests that for malignant cancers, smaller, sub-maximal drug doses may outperform maximum tolerated doses in extending survival Scharovsky et al. (14); Kareva et al. (31); Ledzewicz and Schättler (32); Bajger et al. (33, 34); Bodzioch et al. (35). Modeling CAR-T therapy reveals similar dynamics, emphasizing the potential to optimize dosing intervals and quantities to maximize TTP. Future clinical research should focus on capturing the cumulative immune response to validate these predictions.

### 4.2 Discussion on the sensitivity analysis

Sensitivity analysis underscores the importance of patient-specific parameters in therapy efficacy. Accurate estimation of these parameters before treatment is crucial for personalizing dosing strategies. This aligns with previous immunotherapy studies Kronik et al. (36); Kogan et al. (37). Precise parameter assessment can help determine the optimal dose and interval to maximize therapeutic outcomes. **Figure 12** shows that the tumor growth rate parameter *ρ_T_* significantly impacted the tumor cell population in Equation 2 with initial condition (5), underscoring the role of tumor aggressiveness in disease progression. A high *ρ_T_* indicates rapid tumor growth, making early treatment control more challenging. In later therapy stages, the maximum tumor size parameter *K* became more influential. However, this parameter remains beyond direct therapeutic control.

The dominant factors influencing the CAR-T cell population shifted over time. Initially, the starting CAR-T cell count *C*_0_ was most critical, highlighting the importance of dose selection for effective treatment. As therapy progressed, other parameters became more influential (see **Supplementary Figure S7** in the **Supplementary Material**). After one year, the maximum tumor size *K* had the greatest impact, affecting both CAR-T cell proliferation *ρ_C_* and drug resistance *α_R_*. By day 540, *α_T_*, which controls tumor cell elimination by CAR-T cells, became the key factor. At this stage, the minimal influence of *τ_C_* (CAR-T cell decay rate) reflected the low CAR-T cell count, reducing therapeutic effectiveness. The rising impact of *α_R_* in later stages highlights the need for strategies to overcome drug resistance. Enhancing CAR-T cell survival and expansion in the tumor microenvironment could significantly improve treatment outcomes and support long-term remission. However, it should be marked that this result is not so strong as for the variable *T* because of the dynamic changes in the order of most influential parameters in time; cf. **Supplementary Figure S8** in the **Supplementary Material**.

Sensitivity analysis of Equation 3 with initial conditions (6) and (7) revealed differences in parameter influence on tumor and CAR-T cell dynamics compared to Equation 2 (see **Figure 14**). Both models simulated a single CAR-T cell dose administered at *t* = 0, but in Equation 3, the tumor cell population after 180 and 360 days was most influenced by the CAR-T cell inactivation rate *α_C_*, highlighting the importance of preserving the CAR-T cell population early in therapy to control tumor size. After 540 days, as the CAR-T cell population greatly diminished, **Figure 5**, the tumor growth rate *ρ_T_* became the primary driver of tumor expansion. Since *ρ_T_* reflects patient-specific tumor aggressiveness, targeting it during therapy could be vital for improving outcomes. Over time, the average CART cell production time *τ_C_* became more important, suggesting that prolonging CAR-T cell persistence may enhance tumor control. In contrast, the tumor elimination rate by CAR-T cells *α_T_* was least influential during the first year, highlighting that early therapy challenges are more about maintaining CAR-T cell activation and survival than immediate tumor-killing efficiency. Although initially very important, after 1.5 years, the CAR-T cell proliferation rate *ρ_C_* became the least significant factor, likely due to the decline in the CAR-T cell population and its resulting minimal impact on tumor control.

The CAR-T cell population after 180 days was most influenced by *g_T_*, the tumor half-saturation parameter, which correlates with tumor immunogenicity (higher values indicate a more effective immune response across tumor sizes, see **Supplementary Figure S11** in **Supplementary Material**). After 360 days, *τ_C_* became the key parameter, reflecting the importance of CART cell viability. After 540 days, *ρ_C_* emerged as the most influential factor, underscoring the role of CAR-T cell regeneration for sustained therapeutic efficacy. The least significant parameter after 540 days was *α_T_*, as in the first approach, indicating that tumor elimination remained essential regardless of the treatment strategy. These scenarios require further in silico and *in vivo* investigation to explore how less aggressive tumors may impact CAR-T therapy dynamics. Future studies could also test model-predicted dosing regimens, which may enhance outcomes, as suggested by previous research Kronik et al. (36); Kogan et al. (37).

### 4.3 Conclusions

In conclusion, we emphasize that the proposed mathematical models successfully reproduce the clinical trial results described by Migliorini et al. (3). Additionally, these models enable the exploration of various treatment protocols prior to initiating clinical trials. However, we acknowledge the limitations of our models, primarily stemming from simplifying assumptions. First, our models account for only two components of the immune response – tumor cells and CAR-T cells – whereas real immune reactions involve numerous, more complex interactions. Second, the tumor growth function is assumed to be logistic, but with appropriate clinical data for glioblastoma, the growth function should be fitted to those data, possibly leading to a different type of growth model. Third, the models do not incorporate spatial considerations, which could capture exogenous tumor heterogeneity influenced by factors such as oxygen and glucose supply.

Endogenous factors, such as epigenetics and therapy sensitivity (e.g., related to antigen exposure), further contribute to tumor heterogeneity, which is not addressed in our current approach. In the context of heterogeneity, tumor resistance can be described in greater detail by incorporating tumor cells with varying resistance properties.

Moreover, the models require improved calibration, as the results presented here are qualitative. Incorporating patient-specific data could enhance the models’ accuracy, enabling quantitative predictions and broader clinical applicability.

In the context of personalized medicine, greater efforts should be made to better specify patient-specific model parameters. Thus far, we have conducted sensitivity analysis only for a single-dose regimen, while our results underscore the effectiveness of multiple-dose protocols. Therefore, future work should include sensitivity analysis tailored to multiple dose regimens. Additionally, the modeling of resistance to therapy could be enhanced. For instance, including the delayed triggering of the resistance or incorporate a division of the tumor population into subpopulations of sensitive and resistant cells, allowing for a more accurate representation of tumor heterogeneity and treatment response.

Despite the limitations discussed, we hope that our findings will contribute to the improved application of CAR-T therapy in treating gliomas and other solid tumors. The proposed models are highly versatile and can be adapted to suit specific cases, offering a valuable framework for exploring and optimizing treatment strategies.

## Data Availability

The original contributions presented in the study are included in the article/**Supplementary Material**. Further inquiries can be directed to the corresponding author.

## References

[1] Wu W Klockow JL Zhang M Lafortune F Chang E Jin L. Glioblastoma multiforme (GBM): An overview of current therapies and mechanisms of resistance. Pharmacol Res. (2021) 171:105780. doi: 10.1016/j.phrs.2021.105780 34302977 PMC8384724

[2] Dewdney B Jenkins MR Best SA Freytag S Prasad K Holst J. From signalling pathways to targeted therapies: unravelling glioblastoma’s secrets and harnessing two decades of progress. Signal Transduction Targeted Ther. (2023) 8:400. doi: 10.1038/s41392-023-01637-8 PMC10587102 37857607

[3] Migliorini D Dietrich P-Y Stupp R Linette GP Avery D. Posey J June CH. CAR T-cell therapies in glioblastoma: A first look. Clin Cancer Res. (2018) 24:535 –540. doi: 10.1158/1078-0432 29158268

[4] Eshhar Z Waks T Gross G Schindler D. Specific activation and targeting of cytotoxic lymphocytes through chimeric single chains consisting of antibody-binding domains and the *γ* or *ζ* subunits of the immunoglobulin and T-cell receptors. Proc Natl Acad Sci United States America. (1993) 90:720–4. doi: 10.1073/pnas.90.2.720 PMC45737 8421711

[5] Wrona E Potemski P. A novel immunotherapy — the history of CAR T-cell therapy. Oncol Clin Pract. (2019) 15 202 –207. doi: 10.5603/OCP.2019.0016

[6] Kufel J Lewandowski P. *Terapia CAR-T w leczeniu guzów litych – zastosowanie i ograniczenia (in Polish)* (Starowa Góra, Poland: Wydawnictwo Naukowe ArchaeGraph). (2023).

[7] Cohen AD Garfall AL Stadtmauer EA Melenhorst JJ Lacey SF Lancaster E. B cell maturation antigen–specific CAR T cells are clinically active in multiple myeloma. J Clin Invest. (2019) 129:2210–21. doi: 10.1172/JCI126397 PMC6546468 30896447

[8] Pasvolsky O Kebriaei P Shah BD Jabbour E Jain N. Chimeric antigen receptor T-cell therapy for adult B-cell acute lymphoblastic leukemia: stateof-the-(C)ART and the road ahead. Blood Adv. (2023) 7:3350–60. doi: 10.1182/bloodadvances.2022009462 PMC10345854 36912764

[9] Park JH Rivière I Gonen M Wang X Sénéchal B Curran KJ. Long-term follow-up of CD19 CAR therapy in acute lymphoblastic leukemia. New Engl J Med. (2018) 378:449–59. doi: 10.1056/NEJMoa1709919 PMC6637939 29385376

[10] León-Triana O Pérez-Martínez A Ramírez-Orellana M Pérez-García VM. Dual-target CAR-Ts with on- and off-tumour activity may override immune suppression in solid cancers: A mathematical proof of concept. Cancers. (2021) 13:703. doi: 10.3390/cancers13040703 33572301 PMC7916125

[11] Bodnar M Foryś U Piotrowska MJ Bodzioch M Romero-Rosales JA Belmonte-Beitia J. On the analysis of a mathematical model of CAR-T cell therapy for glioblastoma: Insights from a mathematical model. Int J Appl Mathematics Comput Sci. (2023) 33:379–94. doi: 10.34768/amcs-2023-002

[12] Bodnar M Piotrowska MJ Bodzioch M Belmonte-Beitia J Foryś U. Dual CAR-T cell therapy for glioblastoma: strategies to cure tumour diseases based on a mathematical model. Nonlinear Dynamics. (2025) 113:1637–66. doi: 10.1007/s11071-024-10258-x

[13] Szafrańska-Łęczycka M Bodnar M Piotrowska MJ Krukowski M Belmonte-Beitia J Foryś U. Influence of time delay on the dynamics of a mathematical model of CAR-T cell therapy with logistic tumor growth. Discrete Continuous Dynamical Systems: Ser B. (2025) 30(11):4498–515. doi: 10.3934/dcdsb.2025073

[14] Scharovsky OG Mainetti LE Rozados VR. Metronomic chemotherapy: changing the paradigm that more is better. Curr Oncol. (2009) 16:7–15. doi: 10.1126/scitranslmed.aac5415 PMC2669231 19370174

[15] Bodnar M Foryś U. Three types of simple DDE’s describing tumor growth. J Biol Syst. (2007) 15:453–71. doi: 10.1142/S0218339007002313

[16] Szymańska Z Skrzeczkowski J Miasojedow B Gwiazda P. Bayesian inference of a non-local proliferation model. R Soc Open Sci. (2021) 8:211279. doi: 10.1098/rsos.211279 34849247 PMC8611353

[17] Gwiazda P Miasojedow B Skrzeczkowski J Szymańska Z. Convergence of the EBT method for a non-local model of cell proliferation with discontinuous interaction kernel. IMA J Numerical Anal. (2023) 43:590–626. doi: 10.1093/imanum/drab102

[18] Elishmereni M Kheifetz Y Shukrun I Bevan G Nandy D McKenzie K. Predicting time to castration resistance in hormone sensitive prostate cancer by a personalization algorithm based on a mechanistic model integrating patient data. Prostate. (2016) 76 48–57. doi: 10.1002/pros.23099 26419619

[19] Foryś U Nahshony A Elishmereni M. Mathematical model of hormone sensitive prostate cancer treatment using leuprolide: A small step towards personalization. PloS One. (2022) 17:1–25. doi: 10.1371/journal.pone.0263648 PMC8846544 35167616

[20] Stein AM Grupp SA Levine JE Laetsch TW Pulsipher MA Boyer MW. Tisagenlecleucel model-based cellular kinetic analysis of chimeric antigen receptor-T cells. CPT: Pharmacometrics Syst Pharmacol. (2019) 8 285–295. doi: 10.1002/psp4.12388 30848084 PMC6539725

[21] Radunskaya A Kim R II TW. Mathematical modeling of tumor immune interactions: a closer look at the role of a pd-l1 inhibitor in cancer immunotherapy. Spora: A J Biomathematics. (2018) 4, 41. doi: 10.30707/SPORA4.1Radunskaya

[22] Ghorashian S Kramer AM Onuoha S Wright G Bartram J Richardson R. Tisagenlecleucel in adult relapsed or refractory diffuse large B-cell lymphoma. Nat Med. (2019) 25:1408 –1414. doi: 10.1038/s41591-019-0549-5 31477906

[23] Qi T McGrath K Ranganathan R Dotti G Cao Y. Cellular kinetics: A clinical and computational review of CAR-T cell pharmacology. Advanced Drug Delivery Rev. (2022) 188:114421. doi: 10.1016/j.addr.2022.114421 PMC9520251 35809868

[24] Haycock G Schwartz G Wisotsky D. Geometric method for measuring body surface area: A height-weight formula validated in infants, children and adults. J Pediatr. (1978) 93:68–6. doi: 10.1016/s0022-3476(78)80601-5 650346

[25] The MathWorks Inc. MATLAB software version: 9.13.0 (r2022b) (Natick, Massachusetts, United States). (2022).

[26] Saltelli A Tarantola S Campolongo F Ratto M. Global sensitivity analysis for importance assessment. John Wiley & Sons, Ltd (2002) p. 31–61. doi: 10.1002/0470870958.ch2

[27] Qian G Mahdi A. Sensitivity analysis methods in the biomedical sciences. Math Biosci. (2020) 323:108306. doi: 10.1016/j.mbs.2020.108306 31953192

[28] Bogdańska MU Bodnar M Piotrowska MJ Murek M Schucht P Beck J. A mathematical model describes the Malignant transformation of low grade gliomas: Prognostic implications. PloS One. (2017) 12:1–24. doi: 10.1371/journal.pone.0179999 PMC5538650 28763450

[29] Swanson K Harpold H Peacock D Rockne R Pennington C Kilbride L. Velocity of radial expansion of contrast-enhancing gliomas and the effectiveness of radiotherapy in individual patients: a proof of principle. Clin Oncol. (2008) 20:301–8. doi: 10.1016/j.clon.2008.01.006 18308523

[30] Woodward DE Cook J Tracqui P Cruywagen G Murray J J.R. EA. A mathematical model of glioma growth: the effect of extent of surgical resection. Cell Proliferation. (1996) 29:269–88. doi: 10.1111/j.1365-2184.1996.tb01580.x 8809120

[31] Kareva I Waxman DJ Lakka Klement G. Metronomic chemotherapy: an attractive alternative to maximum tolerated dose therapy that can activate anti-tumor immunity and minimize therapeutic resistance. Cancer Lett. (2015) 358:100–6. doi: 10.1016/j.canlet.2014.12.039 PMC4666022 25541061

[32] Ledzewicz U Schättler H. Application of mathematical models to metronomic chemotherapy: What can be inferred from minimal parameterized models? Cancer Lett. (2017) 401:74–80. doi: 10.1016/j.canlet.2017.03.021 28323033

[33] Bajger P Bodzioch M Foryś U. Singularity of controls in a simple model of acquired chemotherapy resistance. Discrete Continuous Dynamical Syst - B. (2019) 24:2039–52. doi: 10.3934/dcdsb.2019083

[34] Bajger P Bodzioch M Foryś U. Numerical optimisation of chemotherapy dosage under antiangiogenic treatment in the presence of drug resistance. Math Methods Appl Sci. (2020) 43:10671–89. doi: 10.1002/mma.6958

[35] Bodzioch M Bajger P Foryś U. Angiogenesis and chemotherapy resistance: optimizing chemotherapy scheduling using mathematical modeling. J Cancer Res Clin Oncol. (2021) 147:2281–99. doi: 10.1007/s00432-021-03657-9 PMC8236485 34050795

[36] Kronik N Kogan Y Vainstein V Agur Z. Improving alloreactive CTL immunotherapy for Malignant gliomas using a simulation model of their interactive dynamics. Cancer Immunology Immunotherapy. (2008) 57:425–39. doi: 10.1007/s00262-007-0387-z PMC11030586 17823798

[37] Kogan Y Foryś U Shukron O Kronik N Agur Z. Cellular immunotherapy for high grade gliomas: mathematical analysis deriving efficacious infusion rates based on patient requirements. SIAM J Appl Mathematics. (2010) 70:1953–76. doi: 10.1137/08073740X

