# Supplementary material for: CAR-T cell therapy for glioblastoma: insight from mathematical modeling
Source: Front Immunol. 2025 Jun 18;16:1563829. doi: 10.3389/fimmu.2025.1563829 (PMC12213547; doi:10.3389/fimmu.2025.1563829)
Supplement: Supplementary file 1 [file DataSheet1.pdf]

Supplementary Material

1 MODELS AND THEIRS MATHEMATICAL PROPERTIES

1.1 Model with drug resistance

One of the models we focus on in this article is a mechanistic model that describes the interactions between CAR-T cells (C) and glioblastoma cells (T), incorporating an additional component to account for drug resistance (R). This model is represented by a system of ordinary differential equations:

$$\begin{aligned}\frac{d}{dt}T(t) &= \rho_T T(t)\left(1-\frac{T(t)}{K}\right) - \alpha_T(1-R(t))C(t)T(t),\\ \frac{d}{dt}C(t) &= \left(\frac{\rho_C(1-R(t))T(t)}{g_T+T(t)} - \frac{\alpha_C T(t)}{g_C+C(t)} - \frac{1}{\tau_C}\right)C(t),\\ \frac{d}{dt}R(t) &= \alpha_R C(t)\left(1-R(t)\right),\end{aligned} \tag{S1}$$

with all coefficients being positive constants. To complete the model, we impose standard initial conditions defined as:

$$T(0)=T_0\geq 0, \quad C(0)=C_0\geq 0 \quad \text{and} \quad R(0)=R_0=0. \tag{S2}$$

First, note that the local existence and uniqueness of solutions to Eqs. (S1) for any initial data (S2) follow from the smoothness of the system's right-hand side. Furthermore, the proof of non-negativity and global existence for the T and C coordinates of the solution, even in the presence of the reducing factor $(1-R)$, proceeds analogously to the case without this factor, as established in Bodnar et al. (2023) and Bodnar et al. (2025). Hence, the coordinate C remains non-negative. Moreover, we have $\frac{d}{dt}R(0)=\alpha_R C_0 \geqslant 0$ for the initial data Eqs. (S2), which implies that R is non-decreasing at $t=0$ and, consequently, non-negative for $t\geq 0$. To achieve negative values of R, this coordinate would need to decrease strictly over some interval, which does not occur. Additionally, it follows that $R(t)\leq 1$ because, for any $t\geq 0$ where $R(t)>1$, we have $\frac{d}{dt}R(t)<0$, preventing R from crossing the value 1 if it starts below it. For $R\in[0,1]$, it is straightforward to show (as in Bodnar et al. (2025)) that if $T_0\leq K$, then $T(t)\leq K$ for all $t\geq 0$. Thus, the model

dynamics are examined within the invariant space $\mathcal{D} = \{(T, C, R) \in \mathbb{R}^3 : 0 \leq T \leq K,\, C \geq 0,\, 0 \leq R \leq 1\}$.

Additionally, for Eqs. (S1), there exist infinitely many steady states of the form $(0, 0, \bar{R})$ and $(K, 0, \bar{R})$, where $\bar{R} \in [0, 1]$ within the invariant space $\mathcal{D}$. Steady states of the form $(0, 0, \bar{R})$ are unstable. This instability arises because, in the vicinity of $T = 0$, the dominant term in the first equation is $\rho_T T$, which causes repulsion from $T = 0$. For steady states of the form $(K, 0, \bar{R})$, two cases can be considered:

- If R stabilizes at a level $\bar{R}$ such that the inequality

$$\frac{\rho_C(1 - \bar{R})K}{g_T + K} < \frac{1}{\tau_C}$$

  holds, then the coordinate C decreases over time, at least for sufficiently large $t > 0$, leading C to approach 0. Consequently, the variable T approaches its maximum value, K.
- If the derivative of C changes sign over time, the behavior of C can be more complex. However, asymptotically, the dynamics of the model are determined by the limiting value $\bar{R}$. In this case, the behavior mirrors that of the two-dimensional model (see Bodnar et al. (2025) and Szafrańska-Łęczycka et al. (2025)), with the parameters α_T and ρ_C effectively reduced by the factor $(1 - \bar{R})$.

The dynamics of model Eqs. (S1) with the reference parameter values from Table1 (main text) and varying α_R are illustrated in Figure S1 and Figure S2. We observe that, for different values of α_R, the variable R – which reflects the strength of resistance – stabilizes at different levels. Specifically, as α_R increases, the steady-state level of R also increases. For the reference parameter set (with only α_R varying), R stabilizes rapidly, leading to a decline in CAR-T cell count and, ultimately, to tumor regrowth. However, for smaller values of α_R, the CAR-T cell population initially increases, with higher peaks for smaller α_R, see Figure S1. In contrast, for α_R values around 10^{-10}, the CAR-T cell count decreases over time, see Figure S2. Unsurprisingly, the final tumor cell count is lower for smaller α_R values, as seen in both Figure S1 and Figure S2.

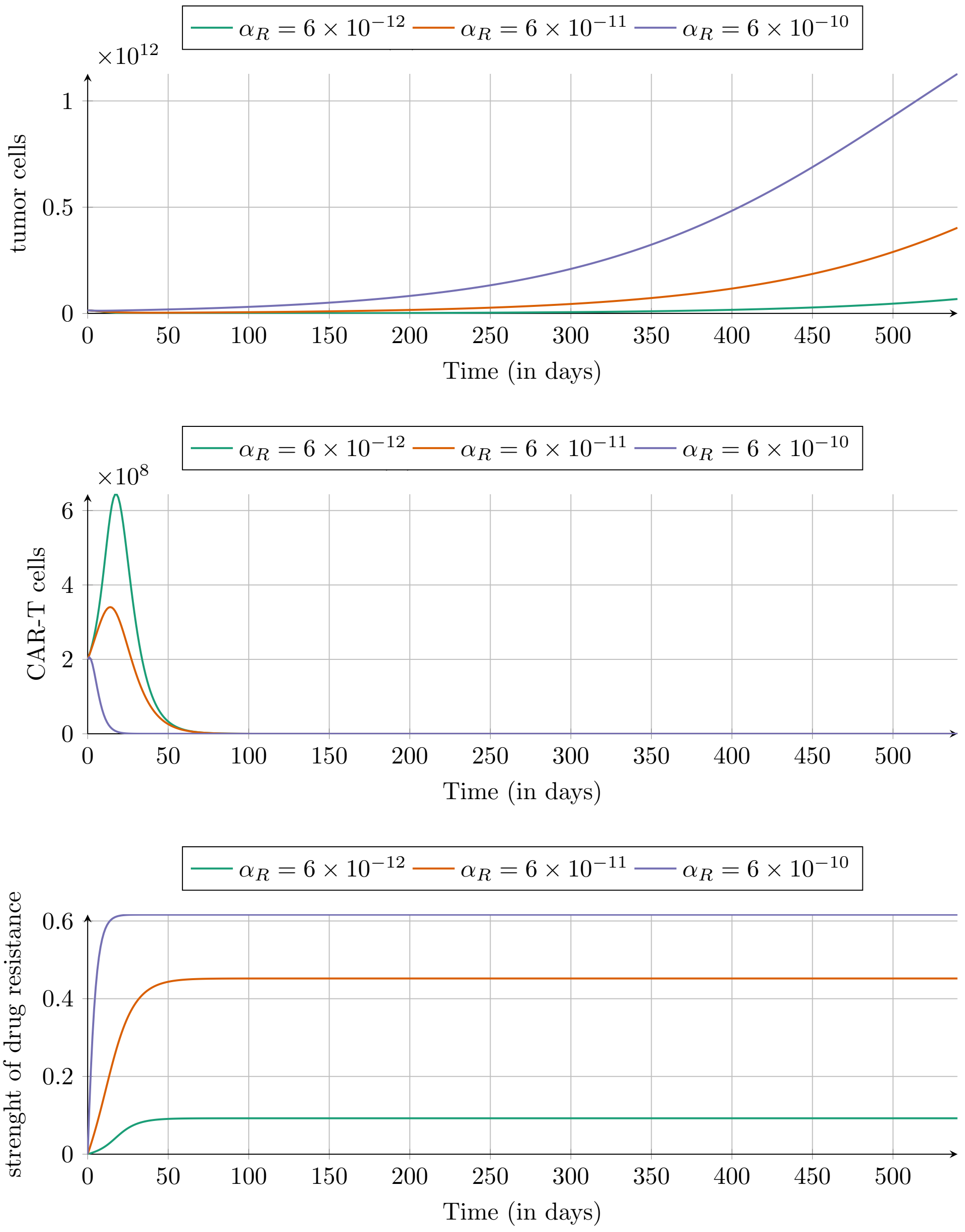

Figure S1: Influence of resistance on model dynamics, i.e., solutions of Eqs. (S1) for α_R equal to 6×10^{-12}, 6×10^{-11}, and 6×10^{-10}, as indicated in the legend. Colors represent the magnitude of α_R, and the vertical axis represents time. Simulation parameters are listed in Table1 (main text), except for the initial conditions: $T_0 = 1.5 \times 10^{10}$ and $C_0 = 2 \times 10^{8}$.

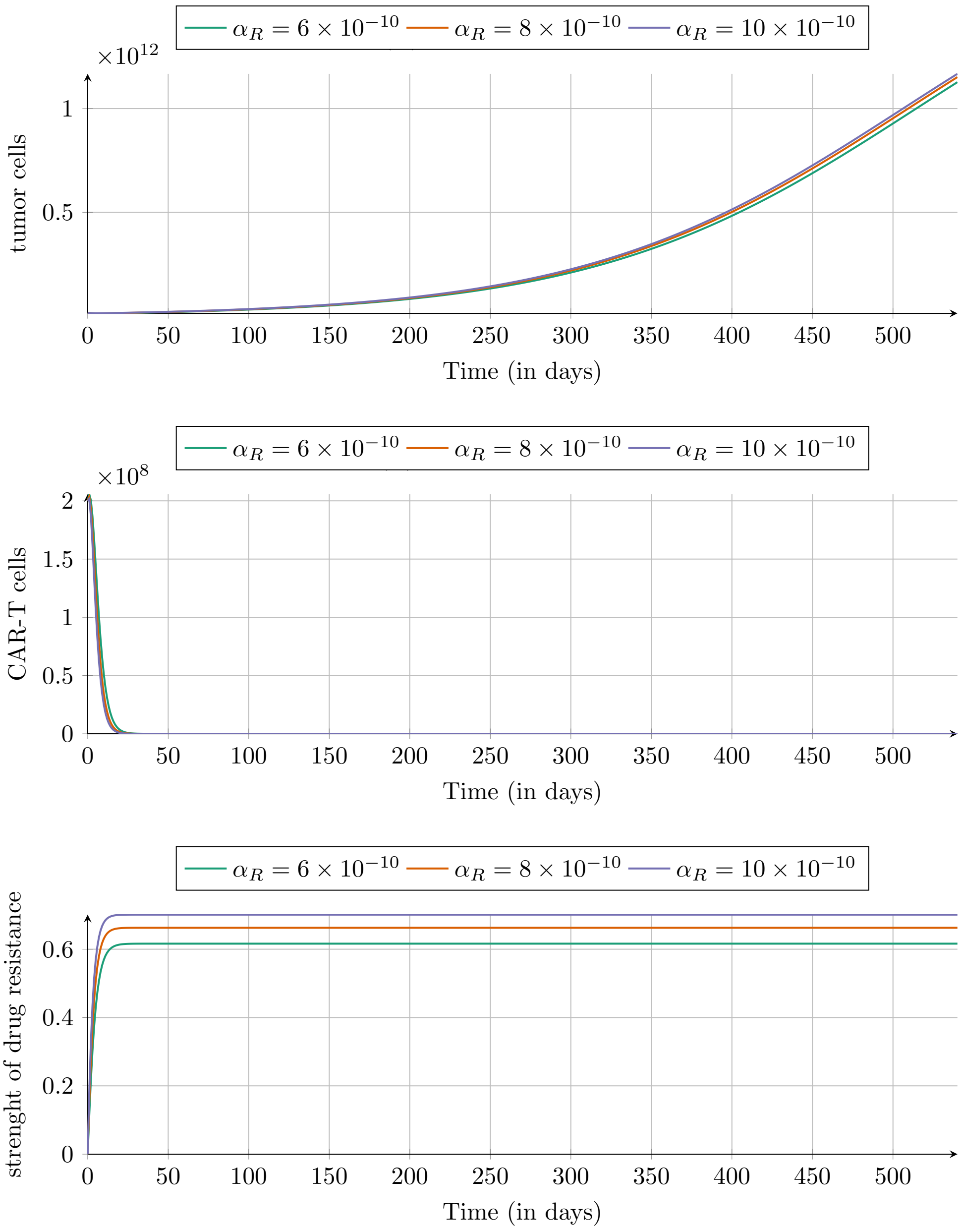

Figure S2: Influence of resistance on model dynamics, i.e., solutions of Eqs. (S1) for α_R equal to 6×10^{-10}, 8×10^{-10}, and 10×10^{-10}, as indicated in the legend. Colors represent the magnitude of α_R, and the vertical axis represents time. Simulation parameters are listed in Table 1 (main text), except for the initial conditions: $T_0 = 1.5 \times 10^{10}$ and $C_0 = 2 \times 10^{8}$.

1.2 Model with time delay

Another mechanistic model describing interactions between CAR-T cells (C) and glioblastoma multiforme cells (T), considered in this paper, is formulated as a system of delay differential equations of the form:

$$\begin{aligned}
\frac{d}{dt}T(t) &= \rho_T\left(1-\frac{T(t)}{K}\right)T(t)-\alpha_T C(t)T(t),\\
\frac{d}{dt}C(t) &= \rho_C\frac{T(t-\tau)C(t-\tau)}{g_T+T(t-\tau)}-\left(\frac{\alpha_C T(t)}{g_C+C(t)}+\frac{1}{\tau_C}\right)C(t),
\end{aligned} \tag{S3}$$

where, t is the independent variable representing time, τ denotes the delay period before the body begins producing active CAR-T cells, and all the model parameters are positive.

For the purpose of qualitative mathematical analysis of Eqs. (S3), we employ non-dimensional variables

$$\hat{t}=\frac{t}{\tau_C},\quad \hat{T}(\hat{t})=\frac{T(t)}{g_T},\quad \hat{C}(\hat{t})=\frac{C(t)}{g_C},$$

which leads to non-dimensional system

$$\begin{aligned}
\frac{d}{d\hat{t}}\hat{T}(\hat{t}) &= \left(\hat{\rho}_T\left(1-\frac{\hat{T}(\hat{t})}{\hat{K}}\right)-\hat{\alpha}_T\hat{C}(\hat{t})\right)\hat{T}(\hat{t}),\\
\frac{d}{d\hat{t}}\hat{C}(\hat{t}) &= \frac{aT(\hat{t}-\hat{\tau})\hat{C}(\hat{t}-\hat{\tau})}{1+\hat{T}(\hat{t}-\hat{\tau})}-\left(\frac{b\hat{T}(\hat{t})}{1+\hat{C}(\hat{t})}+1\right)\hat{C}(\hat{t}),
\end{aligned} \tag{S4}$$

where

$$\begin{aligned}
\hat{\rho}_T=\rho_T\tau_C,\quad \hat{\alpha}_T=\alpha_T g_C\tau_C,\quad \hat{\tau}=\frac{\tau}{\tau_c},\\
a=\rho_C\tau_C,\quad b=\frac{\alpha_C g_T\tau_C}{g_C},\quad \hat{K}=\frac{K}{g_T}\,.
\end{aligned}$$

In the following, we use these non-dimensional variables and parameters, written without hats, to simplify the notation.

For Eqs. (S4), we need to define initial data as functions on the interval $[-\tau, 0]$. To reflect the fact that there is no treatment for $t<0$, while an influx of C_0 CAR-T cells occurs at

$t = 0$, these functions are given by

$$T(t) = \frac{K}{1 - \left(1 - \frac{K}{T_0}\right) \mathrm{e}^{-\rho_T t}}, \text{ for } t \in [-\tau, 0],$$

and (S5)

$$C(t) = \begin{cases} 0, & \text{for } t \in [-\tau, 0), \\ C_0 > 0, & \text{for } t = 0. \end{cases}$$

The analytical results concerning the global existence and uniqueness of positive solutions to Eqs. (S4) with general initial data, including Eqs. (S5), as well as the analysis of system dynamics and the existence of bifurcations, are presented in Szafrańska-Łęczycka et al. (2025). Here, we provide illustrative examples of how time delay influences model dynamics, using the reference parameter values listed in Table1 (main text). Figure S3 and Figure S4 show the solutions of Eqs. (S3) with initial data given by Eqs. (S5), for two different sets of T_0 and C_0, and varying τ. In Figure S3, with smaller initial values of T_0 and C_0, we observe that larger delays result in a smaller initial expansion of CAR-T cells, but a slower decline afterward – yielding a slightly lower CAR-T cell count at the end of the simulation. Due to the high initial CAR-T cell count, the tumor initially shrinks (with small oscillations at large delays) and then regrows. In Figure S4, a more typical delay – dependent behavior is observed, with both variables exhibiting oscillations. For larger delays, the peak in CAR-T cell dynamics is smaller, which leads to a higher final tumor cell count. Interestingly, in both cases, the final tumor size for the smallest delay appears to fall between the other outcomes, possibly due to the complex interplay between the initial conditions and the time delay (as larger delays enhance the backward influence of logistic tumor growth).

Note that all previous analyses address the case of a single injection of CAR-T cells at time $t = 0$ within the model. In this work, we focus on the scenario of periodic treatment. Specifically, we prove the existence of solutions and the local stability of periodic solutions for the impulsive Eqs. (S4)–(S6). To achieve this, we assume that a dose of m CAR-T cells is administered at each time point $t_n = nP$, where $P > \tau$ is a given positive constant defining the interval between successive CAR-T cell applications. During each interval $[t_n, t_{n+1})$, the variables T and C satisfy Eqs. (S4), and

$$T(t_n) = \lim_{t \to t_n^-} T(t), \qquad C(t_n) = \lim_{t \to t_n^-} C(t) + m. \tag{S6}$$

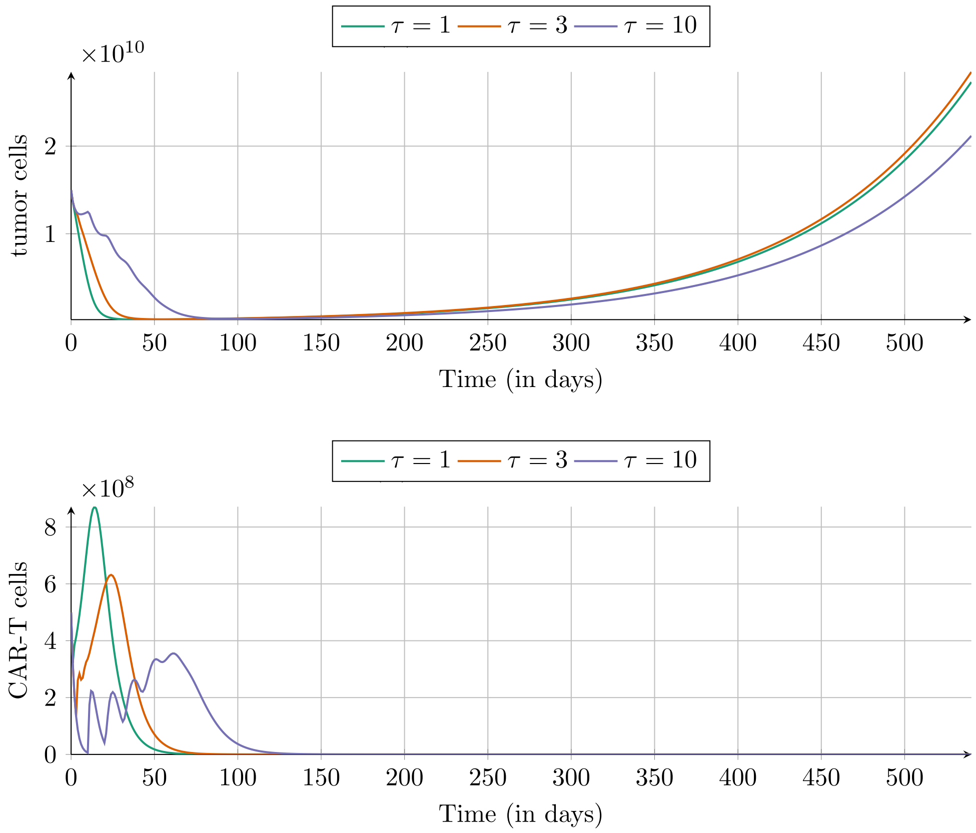

Figure S3: The impact of time delay on model dynamics, i.e., solutions of Eqs. (S3) with initial conditions (S5) for τ equal to 1, 3, and 10, as indicated in the legend. Colors represent the magnitude of the delay, and the vertical axis represents time. Simulation parameters are listed in Table 1 (main text), except for $T_0 = 1.5 \times 10^{10}$ and $C_0 = 5 \times 10^8$.

THEOREM 1. *Assume that* $T(t) = 0$ *for* $t \in [-\tau, 0]$. *Let* $t \in [nP, (n+1)P)$, $n \in \mathbb{N}$, *and define*

$$C^*(t) = \frac{m}{1 - \mathrm{e}^{-P}} \mathrm{e}^{-(t-nP)} \;\; \textit{for } n \in \mathbb{N}.$$

Then, the pair $(0, C^*(t))$ *represents a periodic solution of the impulsive Eqs.* (S4)–(S6)*, which is locally stable for*

$$\rho_T P < m\alpha_T.$$

PROOF. Consider $T(t) \equiv 0$, which clearly satisfies Eqs. (S4) for the initial condition $T(t) = 0$ for $t \in [-\tau, 0]$. Then, for the variable C, we obtain an impulsive equation of the

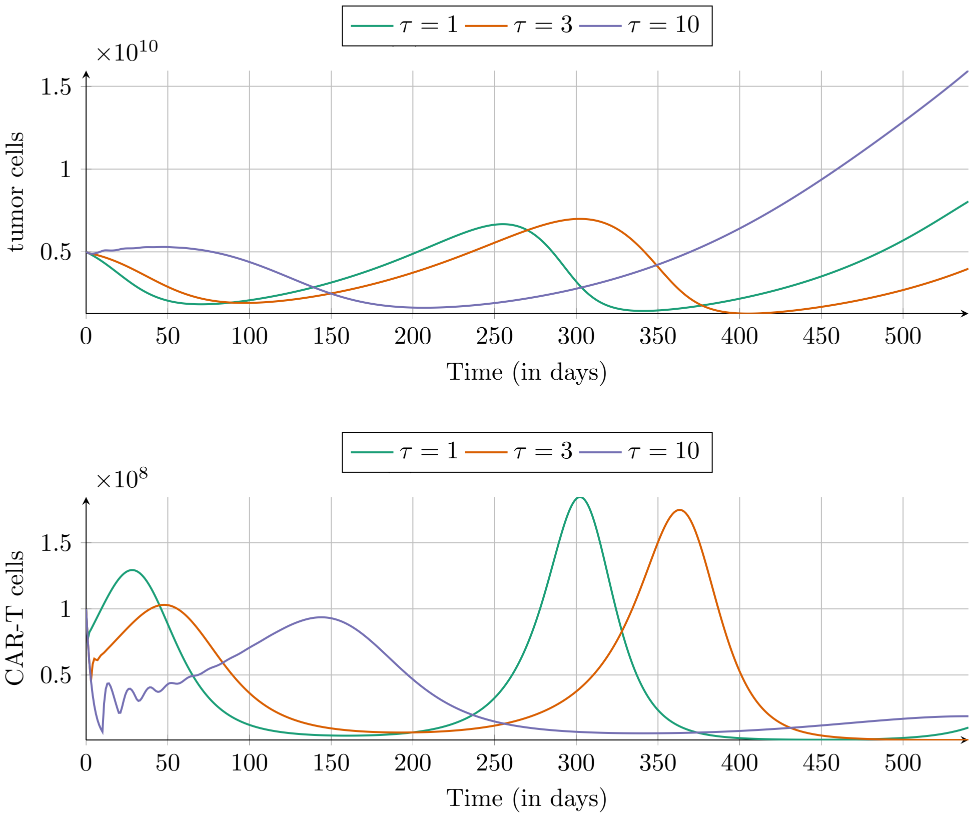

Figure S4: The impact of time delay on model dynamics, i.e., solutions of Eqs. (S3) with initial conditions (S5) for τ equal to 1, 3, and 10, as indicated in the legend. Colors represent the magnitude of the delay, and the vertical axis represents time. Simulation parameters are listed in Table 1 (main text), except for $T_0 = 0.5 \times 10^{10}$ and $C_0 = 1 \times 10^8$.

form

$$\begin{aligned} \frac{d}{dt}C &= -C, \quad \text{for } t \in [nP, (n+1)P), \\ C(nP) &= \lim_{t \to t_n^-} C(t) + m, \quad t_n = nP, \ n \geq 1. \end{aligned} \tag{S7}$$

It is obvious, that $C^*(t)$ satisfies $\frac{d}{dt}C = -C$. In addition, we get

$$\lim_{t \to t_n^-} C^*(t) + m = \frac{m\mathrm{e}^{-P}}{1 - \mathrm{e}^{-P}} + m = \frac{m}{1 - \mathrm{e}^{-P}} = C^*(nP), \quad n \geq 1.$$

Hence, C^* satisfies Eqs. (S7) and, consequently $(0, C^*)$ is a solution of Eqs. (S4)–(S6).

To verify the local stability of this periodic solution, we expand an arbitrary solution around it. We obtain

$$T = \epsilon T_1 + O(\epsilon^2),\ \ C = C^* + \epsilon C_1 + O(\epsilon^2),\ \ \epsilon \ll 1.$$

Let us consider the system of the first-order approximation

$$\begin{aligned} \frac{d}{dt}T_1(t) &= \big(\rho_T - \alpha_T C^*(t)\big)T_1(t), \\ \frac{d}{dt}C_1(t) &= aC^*(t-\tau)T_1(t-\tau) - \frac{bC^*(t)}{1+C^*(t)}T_1(t) - C_1(t). \end{aligned} \tag{S8}$$

Note that the first equation in Eqs. (S8) ensures the non-negativity of the T_1 variable for non-negative initial conditions, analogous to the first equation in Eqs. (S4). Assume that $T_1(0) > 0$, then $T_1(t) > 0$ for any $t > 0$. To demonstrate the local stability of the periodic solution, we first prove that $T_1(t) \to 0$ as $t \to \infty$. For this purpose, we calculate

$$\int\limits_{T_1(0)}^{T_1(t)} \frac{dT_1}{T_1} = \int\limits_0^t \big(\rho_T - \alpha_T C^*(s)\big)ds.$$

Thus,

$$\ln\frac{T_1(t)}{T_1(0)} = \int\limits_0^t \big(\rho_T - \alpha_T C^*(s)\big)ds.$$

Let $t \in [nP, (n+1)P)$. Note, that

$$\int\limits_0^t C^*(s)ds = \int\limits_0^{nP} C^*(s)ds + \int\limits_{nP}^t C^*(s)ds.$$

Additionally, the periodicity of C^* implies

$$\int\limits_0^{nP} C^*(s)ds = n\int\limits_0^{P} C^*(s)ds.$$

Simple calculations yield

$$\int_{nP}^{t} C^*(s)ds = \int_{nP}^{t} \frac{m\mathrm{e}^{-(s-nP)}}{1-\mathrm{e}^{-P}} ds = \frac{m\left(1-\mathrm{e}^{-(t-nP)}\right)}{1-\mathrm{e}^{-P}} \quad \Longrightarrow \quad \int_{0}^{P} C^*(s)ds = m.$$

Thus,

$$\begin{aligned}
\ln\frac{T_1(t)}{T_1(0)} &= \int_{0}^{nP} \big(\rho_T - \alpha_T C^*(s)\big) ds + \int_{nP}^{t} \big(\rho_T - \alpha_T C^*(s)\big) ds \\
&= \rho_T t - \alpha_T n m - \alpha_T \frac{m\left(1-\mathrm{e}^{-(t-nP)}\right)}{1-\mathrm{e}^{-P}} \\
&= n\left(\rho_T P - \alpha_T m\right) + \rho_T(t-nP) + \alpha_T \frac{m\left(\mathrm{e}^{-(t-nP)}-1\right)}{1-\mathrm{e}^{-P}}.
\end{aligned}$$

Eventually,

$$\begin{aligned}
0 \leqslant T_1(t) &\le T_1(0)\exp\left(n\big(\rho_T P - \alpha_T m\big) + \left(\rho_T P - \frac{m\alpha_T}{1-\mathrm{e}^{-P}}\right)\right) \\
&= D\mathrm{e}^{n(\rho_T P - \alpha_T m)} = D\mathrm{e}^{-nA}, \quad \text{where } A = \alpha_T m - \rho_T P, \;\; D = \text{const}.
\end{aligned}$$

Moreover, for $t \in [nP, (n+1)P)$ we have $n+1 > \frac{t}{P}$, and therefore

$$T_1(t) \le D\mathrm{e}^{-nA} = D_1\mathrm{e}^{-(n+1)A} \le D_1\mathrm{e}^{-tB}, \quad D_1 = D\mathrm{e}^{A},\; B = \frac{A}{P} > 0,$$

which gives the estimation independent on $n \in \mathbb{N}$. Hence, to have $T_1(t) \to 0$ for $t \to \infty$, it is sufficient to assume

$$A > 0 \iff \rho_T P < m\alpha_T.$$

Now, under this assumption, we show that $|C_1(t) - C^*(t)| \to 0$ for $t \to \infty$.
Let $W(t) := C_1(t) - C^*(t)$, then

$$\frac{d}{dt}W(t) = aC^*(t-\tau)T_1(t-\tau) - \frac{bC^*(t)}{1+C^*(t)}T_1(t) - W(t).$$

Note, that non-negativity of C^* and T_1 yields

$$-\frac{bC^*(t)}{1+C^*(t)}T_1(t) - W(t) \leq \frac{d}{dt}W(t) \leq aC^*(t-\tau)T_1(t-\tau) - W(t). \tag{S9}$$

Next, we multiply both sides of (S9) by e^t and rewrite it in the following way

$$-\frac{bC^*(t)D_1\mathrm{e}^{(1-B)t}}{1+C^*(t)} \leq \frac{d}{dt}W(t)\mathrm{e}^t + W(t)\mathrm{e}^t \leq aC^*(t-\tau)D_1\mathrm{e}^{B\tau}\mathrm{e}^{(1-B)t},$$

and defining $D_2 = bD_1$, $D_3 = aC^*_{\max}D_1\mathrm{e}^{B\tau} = \frac{amD_1\mathrm{e}^{B\tau}}{1-\mathrm{e}^{-P}}$, we obtain

$$-D_2\mathrm{e}^{(1-B)t} \leq \frac{d}{dt}\Big(W(t)\mathrm{e}^t\Big) \leq D_3\mathrm{e}^{(1-B)t}.$$

Integration from 0 to t yields

$$-D_2\int_0^t \mathrm{e}^{(1-B)s}ds \leq \int_0^t \frac{d}{ds}\Big(W(s)\mathrm{e}^s\Big)ds \leq D_3\int_0^t \mathrm{e}^{(1-B)s}ds.$$

Hence,

$$\left(W(0) + \frac{D_2}{1-B}\left(1-\mathrm{e}^{(1-B)t}\right)\right)\mathrm{e}^{-t} \leq W(t) \leq \left(W(0) + \frac{D_3}{B-1}\left(1-\mathrm{e}^{-Bt}\right)\right)\mathrm{e}^{-t}.$$

The above inequalities imply that $|C_1(t) - C^*(t)| \to 0$ as $t \to +\infty$. This demonstrates that the periodic solution is locally stable for $\rho_T P < m\alpha_T$, completing the proof.

2 SUPPLEMENTARY RESULTS ON THE EFFECTIVENESS OF TREATMENT PROTOCOLS

Figure S5 illustrates the dynamics of the tumor and CAR-T cell populations under the **second approach** to treatment, involving the administration of seven equal doses of CAR-T cells. Each dose, including the initial dose, was 7.23×10^7, 1×10^8, or 1.89×10^8 cells, administered at 15-week intervals. Each administration of CAR-T cells initially reduced the tumor cell population, followed by oscillatory behavior. The most pronounced effect was observed with the lowest dose 7.23×10^7, which delayed tumor regrowth to its initial size for the longest period. Comparable results were obtained with the medium dose 1×10^8.

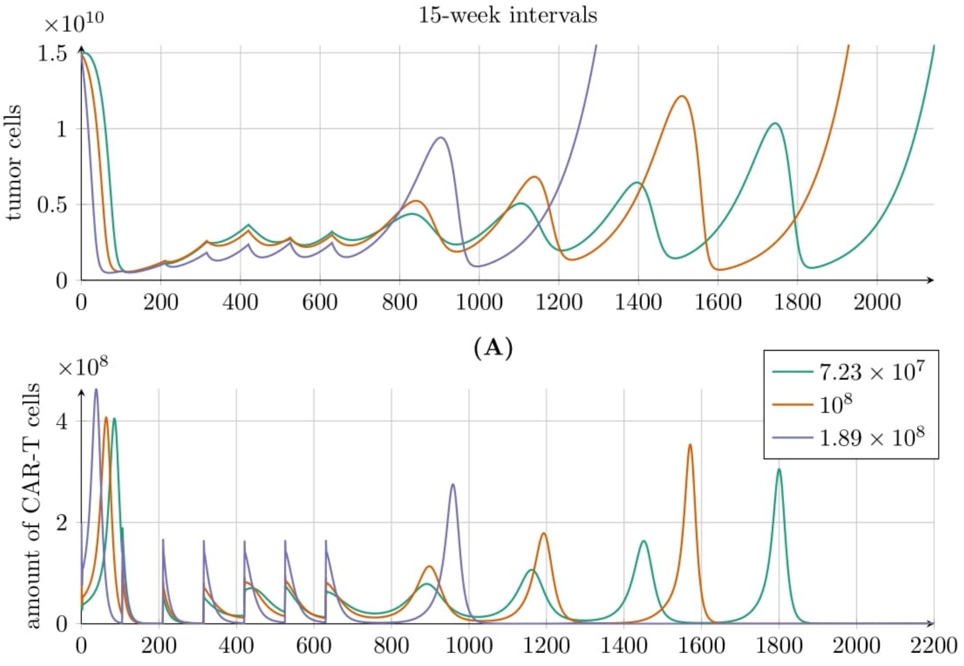

(A)

(B)

Figure S5: Tumor (panel A) and CAR-T cell (panel B) population dynamics modeled by Eqs. (S3), with initial conditions given in (S5), under the **second approach** Migliorini et al. (2018). Seven equal doses of HER2/CMV CAR-T cells were administered at 15-week intervals, with individual dose sizes of 7.23×10^7 (green line), 1×10^8 (red line), and 1.89×10^8 (purple line) cells. Simulations assume a 70 kg, 180 cm patient. Parameters and initial conditions are provided in Table 1 (in the main text), except for C_0, which matches the dose size for each of the three scenarios.

In contrast, the highest dose 1.89×10^8 led to the fastest tumor regrowth and the shortest duration of oscillatory behavior.

The CAR-T cell population also exhibited oscillatory dynamics, with two to five immune responses observed depending on the dose. Interestingly, the two lower doses resulted in a higher frequency and longer duration of immune responses, indicating a sustained immune engagement with the tumor. Notably, the lowest dose demonstrated the longest cumulative therapeutic effect, with the CAR-T cell population persisting for approximately 1 800 days. A similar behavior was observed for a 10-week interval, as shown in Figure 11 of the main text.

3 SENSITIVITY ANALYSIS

For Eqs. (S1) and (S3), we utilize the Morris method (see Qian and Mahdi (2020)) for sensitivity analysis. This method assesses the sensitivity of a specific model output, f, by examining the elementary effects of variations in the input parameters. The algorithm operates as follows:

1. **Re-scaling Input Parameters.** We linearly rescale the parameters to the interval $[0, 1]$. For each model parameter p_i, $i = 1, \ldots, 11$, we define a new parameter $k_i \in [0, 1]$, and set
$$k_i = \frac{p_i - p_{i,\min}}{p_{i,\max} - p_{i,\min}},$$
where $p_{i,\min}$ and $p_{i,\max}$ denote the minimum and maximum values of the parameter p_i, respectively. This normalization ensures that all parameters are rescaled to a comparable range, facilitating analysis on a unified scale.
2. **Generating a Net of Points.** For a fixed $n \in \mathbb{N}$, we divide each of the 11 intervals $[0, 1]$ into $n - 1$ equal subintervals, forming a grid of points. Each point of this grid is of the form $\left(\frac{j_1}{n-1}, \ldots, \frac{j_{11}}{n-1}\right)$, where $j_i \in \{0, 1, \ldots, n-1\}$. This approach ensures uniformly spaced values for each input parameter, providing comprehensive coverage of the input space. In our analysis, we set $n = 21$.
3. **Generating Starting Points.** We set $\Delta = \frac{3}{n-1}$ and randomly choose M initial points (one for each iteration) from the previously generated grid, ensuring that for any selected initial point, $j_i \leq n - 3$. This random selection provides diverse starting points for further analysis, while the condition on j_i ensures sufficient "space" for subsequent calculations.
4. **Calculating Elementary Effects.** For any $m \in \{1, 2, \ldots, M\}$, where M is the number of iterations, we take the previously chosen initial set of parameters $k = (k_1, \ldots, k_{11})$. A random permutation π of the set $\{1, 2, \ldots, 11\}$ is selected. The elementary effect is then calculated as follows
$$EE_m(k_{\sigma(i)}) = \frac{f(k + \Delta e_{\sigma(i)}) - f(k)}{\Delta},$$
which helps us estimate how changes in specific parameters affect the specific model output. Next, we update k to $k + \Delta e_{\sigma(i)}$, increment i to $i + 1$, and calculate the next elementary effect for the updated $k_{\sigma(i)}$.

5. **Calculating the Moments of the Distribution of Elementary Effects.** For each input parameter ($i = 1, \ldots, 11$), the moments are computed as follows

$$\mu_i = \sum_{m=1}^{M} \frac{EE_m(k_i)}{M}, \quad \mu_i^* = \sum_{m=1}^{M} \left| \frac{EE_m(k_i)}{M} \right|, \quad \sigma_i = \sqrt{\sum_{m=1}^{M} \frac{(EE_m(k_i) - \mu_i)^2}{M}},$$

where:

- $EE_m(k_i)$ is the elementary effect for the i-th input parameter in the m-th iteration,
- μ_i measures the average effect of the i-th parameter,
- μ_i^* measures the total effect of the i-th parameter—a high value indicates a large influence of the parameter while avoiding cancellation effects,
- σ_i estimates the non-linear and interaction effects of the i-th parameter.

The total number of model evaluations required in the Morris method for our models is $12M$. In our analysis, we set $M = 1000$.

3.1 Sensitivity Analyzes – supplementary results

Detailed results of the sensitivity analysis for individual variables not included in the main article are presented below. These results illustrate the impact of model parameters and initial values on the size of the modeled cellular populations over a 540-day period. For clarity, the measures μ_i^* and σ_i were normalized to the largest value observed in the analysis. The top plots present the analyzed influence over the entire period, while the bottom plots focus on the first 100 days.

3.1.1 Detailed Results of sensitivity analysis for Eqs. (S1)

Figure S6 illustrates how changes in parameter values and initial conditions influence the simulation outcomes of the tumor cell population according to the **first approach** to treatment Migliorini et al. (2018), involving a single administration of a CAR-T dose modeled by Eqs. (S1) with initial conditions given by Eqs. (S2). For the first 29 days, the initial tumor cell number T_0 had the greatest impact on tumor cell population dynamics. However, from day 29 until day 540, its influence was overtaken by the parameter ρ_T, which describes the tumor growth rate. Notably, over time, the maximum tumor size K became increasingly important, eventually becoming the second most influential parameter from day 400 onward in the sensitivity analysis. The remaining parameters exhibited similar influences on tumor cell population dynamics throughout the entire period. The parameter α_T, which describes the efficient inactivation of CAR-T cells by the tumor, had the smallest

effect on tumor cell population size. This contrasts with its significance in the σ_i plots, where the least influential parameters were T_0 and ρ_T. The remaining parameters exhibited behavior comparable to α_T.

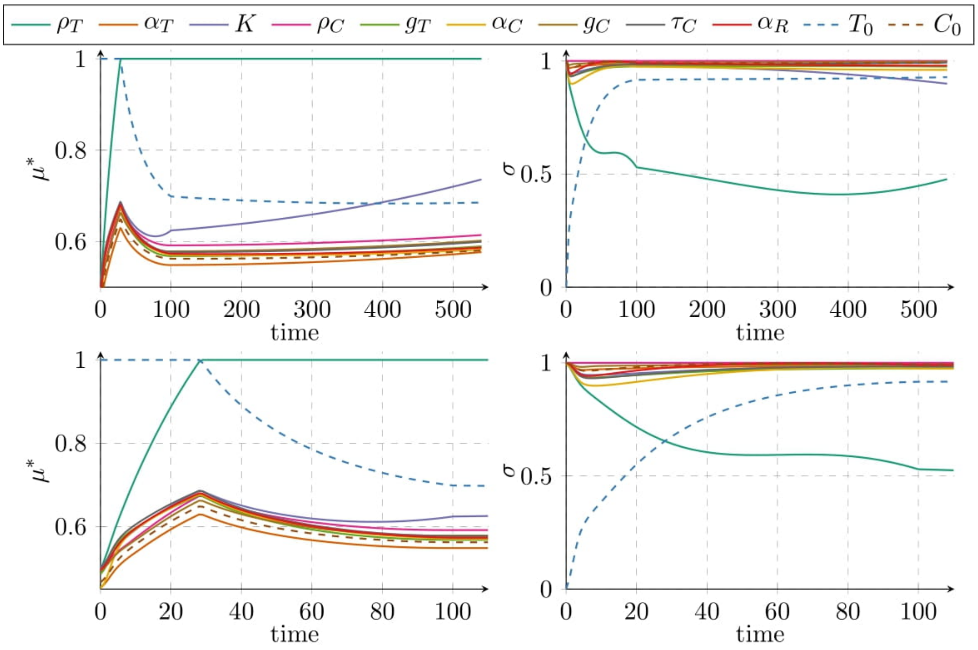

Figure S6: Sensitivity analysis results for the variable T (tumor cell population) under the **first approach** Migliorini et al. (2018), involving a single CAR-T dose modeled by Eqs. (S1) with initial conditions (S2) over the first 540 days of treatment. Colors represent parameters or initial conditions, as shown in the legend. Simulation parameters are listed in Table 1 (main text), with C_0 marked by ($\star$). The μ_i^* plots show the impact of specific model parameters on tumor cell population size, while the σ_i plots highlight parameters driving nonlinear and interactive effects in the model.

We also analyzed the impact of individual parameters and initial conditions on the simulation outcomes of the CAR-T cell population. Figure S7 reveals frequent and significant shifts in the parameters that most influence the values of the μ_i^* and σ_i measures, with different parameters dominating at various periods. For both measures, dominant parameters can be identified only during the first 40 days. For the μ_i^* measure, the most important parameters include the CAR-T proliferation rate ρ_C, the CAR-T inactivation rate α_C, the initial tumor cell number T_0, and the tumor growth rate ρ_T. Between days 5 and 37, the least influential parameter was α_T, which describes the inactivation of CAR-T

cells by the tumor. Beyond the first 40 days, it becomes nearly impossible to determine a clear hierarchy of parameter influence due to their highly dynamic behavior. For the σ_i measure, the most influential parameters during the first 40 days were the initial number of CAR-T cells C_0, the CAR-T proliferation rate ρ_C, the tumor growth rate ρ_T, and the initial tumor cell number T_0. Similar to the μ_i^* measure, α_T remained the least influential parameter during this period. After this initial phase, the dynamic interactions between parameters made it impossible to establish a definitive order of importance for either the μ_i^* or σ_i measures.

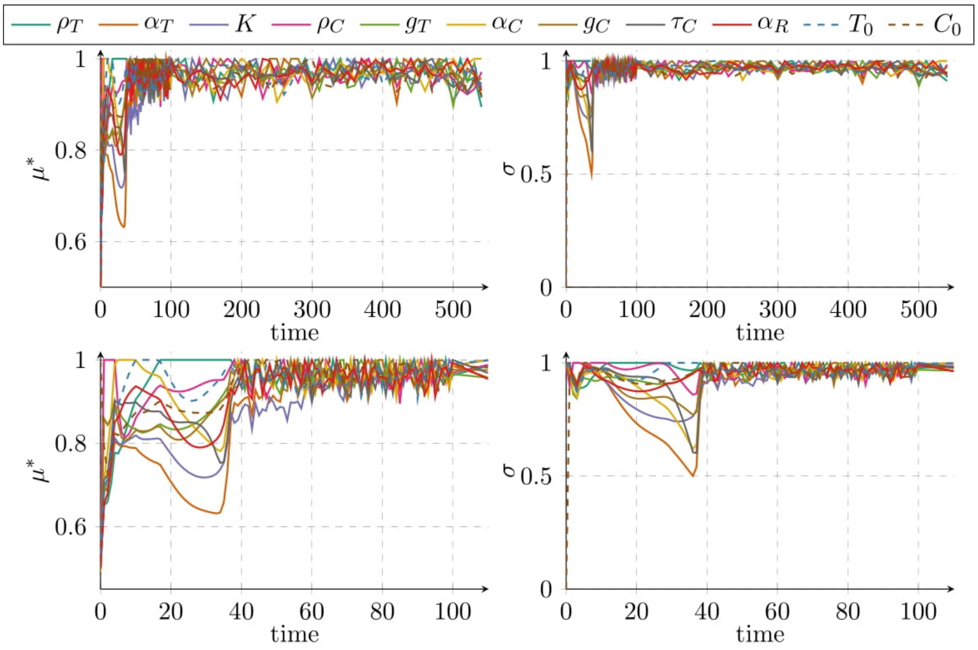

Figure S7: Sensitivity analysis results for the variable C (CAR-T cell population) under the **first approach** Migliorini et al. (2018), involving a single CAR-T dose modeled by Eqs. (S1) with initial conditions (S2) over the first 540 days of treatment. Colors represent parameters or initial conditions, as shown in the legend. Simulation parameters are listed in Table 1 (main text), with C_0 marked by ($\star$). The μ_i^* plots show the impact of specific model parameters on CAR-T cell population size, while the σ_i plots highlight parameters driving nonlinear and interactive effects. The plots reveal highly dynamic shifts in which parameters most influence the μ_i^* and σ_i measures.

Figure S8 presents the exact values of μ^* at three time points – 180, 360, and 540 days – to better assess the impact of individual parameters and initial conditions on the variable C. At 180 days, the initial CAR-T cell count C_0 had the greatest impact, followed by the

maximum tumor size K, while α_T exhibited the least influence. By 360 days, K became the most influential parameter, whereas C_0 and τ_C, representing the mean lifetime of active CAR-T cells at the tumor site, had relatively minor effects. By 540 days, α_T emerged as the most critical factor influencing CAR-T cell population dynamics. At this stage, α_R, reflecting the strength of drug resistance, was the second most influential parameter, while α_C, representing the rate of CAR-T cell inactivation, had the least impact on CAR-T cell population size.

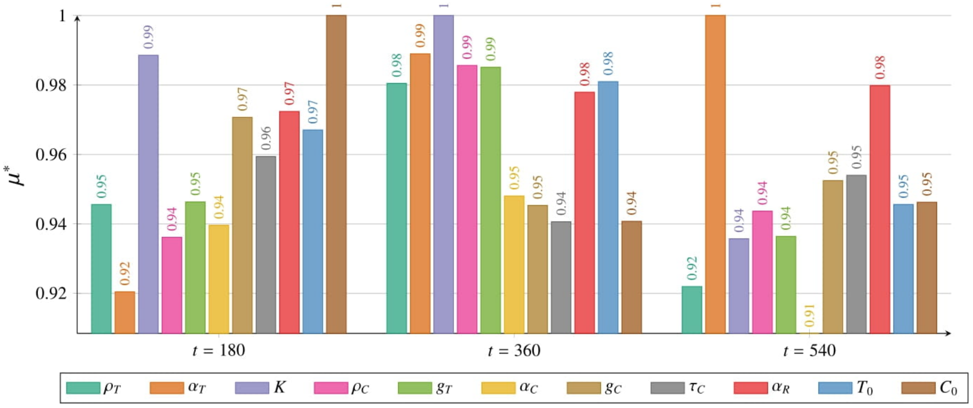

Figure S8: The impact of individual parameters and initial conditions on the CAR-T cell population under the **first approach** Migliorini et al. (2018) with a single CAR-T cell dose, modeled by Eqs. (S1) with initial conditions (S2). Results are displayed for 180 days (left), 360 days (middle), and 540 days (right). Colors represent parameters or initial conditions, as per the legend. The vertical axis depicts the μ_i^* measure. Simulation parameters are in Table 1 (in the main text), with C_0 marked as $(\star)$.

3.1.2 Detailed Results of sensitivity analysis for Eqs. (S3)

Figure S9 illustrates the changes in the tumor cell population based on parameter values and initial conditions under the **third approach** to treatment Migliorini et al. (2018), modeled by Eqs. (S3) with initial conditions (S5). For the first 8 days, the initial tumor cell count T_0 had the greatest influence on the dynamics of the variable T. Then, the initial CAR-T cell count C_0 became the most influential factor. Between days 10 and 29, the CAR-T proliferation rate ρ_C took the leading role. However, from day 30 to day 450, the rate of CAR-T cell inactivation α_C became the dominant parameter. Toward the end of the analyzed period, the tumor half-saturation level g_T and subsequently the tumor growth rate ρ_T emerged as the most influential factors. Notably, the maximum tumor size K consistently had a significant impact on the μ_i^* measure throughout the analysis. Interestingly, from day

30 onward, the initial tumor cell count T_0 and the initial CAR-T cell count C_0 exhibited similar levels of influence. In contrast, α_T, which represents the effective inactivation of CAR-T cells by the tumor, remained the least influential parameter between days 20 and 520. Additionally, the importance of ρ_C declined after day 30, eventually becoming the least influential parameter on tumor cell population size by the end of the analysis. The σ_i graphs provide additional insights. During the first 6 days, C_0 was the most influential parameter, followed by τ, which represents the average time required to trigger CAR-T cell proliferation, for the next 9 days. From days 15 to 75 and again from 450 to 540, α_C had the greatest impact on tumor size, while T_0 became dominant during the intervening period. Notably, T_0 was the least influential parameter during the first 15 days of the analysis. After this point, α_T and the CAR-T half-saturation level g_C consistently showed the smallest impact on the σ_i measure, while the remaining parameters maintained relatively similar levels of influence throughout the analysis.

Figure S10 presents an analogous analysis of the influence of individual parameters and initial conditions on the CAR-T cell population under the **third approach** to treatment Migliorini et al. (2018). The μ_i^* plots reveal frequent shifts in the most influential factor, with this role being assumed at various points by all parameters except the initial tumor cell count T_0 and the maximum tumor size K. Throughout the entire period, the impact of individual parameters was highly dynamic, especially after 400 days. Between days 85 and 450, the parameter α_T, representing the inactivation of tumor cells by CAR-T cells, had the least impact on the CAR-T cell population. The σ_i plots highlight strong interactions between parameters, reflecting patterns similar to those in the μ_i^* plots. As with the μ_i^* results, all parameters except T_0 and α_T emerged as the most influential at certain points. Notably, during the first 5 days, the value of C_0 had the least impact on the σ_i measure. From day 30 to 250 and again from 270 to 375, α_T had the least impact on tumor size, while T_0 took over this role during the intervening period.

Following the methodology outlined in Subsection 2.2.1, we present the μ^* values at three time points – 180, 360, and 540 days – for each parameter and initial condition affecting the variable C. After 180 days, the tumor half-saturation level g_T had the greatest impact on the CAR-T cell population size. The next most influential factors were τ, representing the average time required to trigger CAR-T cell proliferation, the CAR-T half-saturation level g_C, and the initial CAR-T cell count C_0. The remaining parameters—except for α_T, which had the least effect at this stage – exhibited similar impacts on CAR-T cell dynamics. By 360 days, all parameters exerted a stronger influence on the CAR-T cell population. The most significant parameter at this point was the mean lifetime of active CAR-T cells at the tumor site, τ_C, followed closely by g_C and τ. Most of the remaining parameters

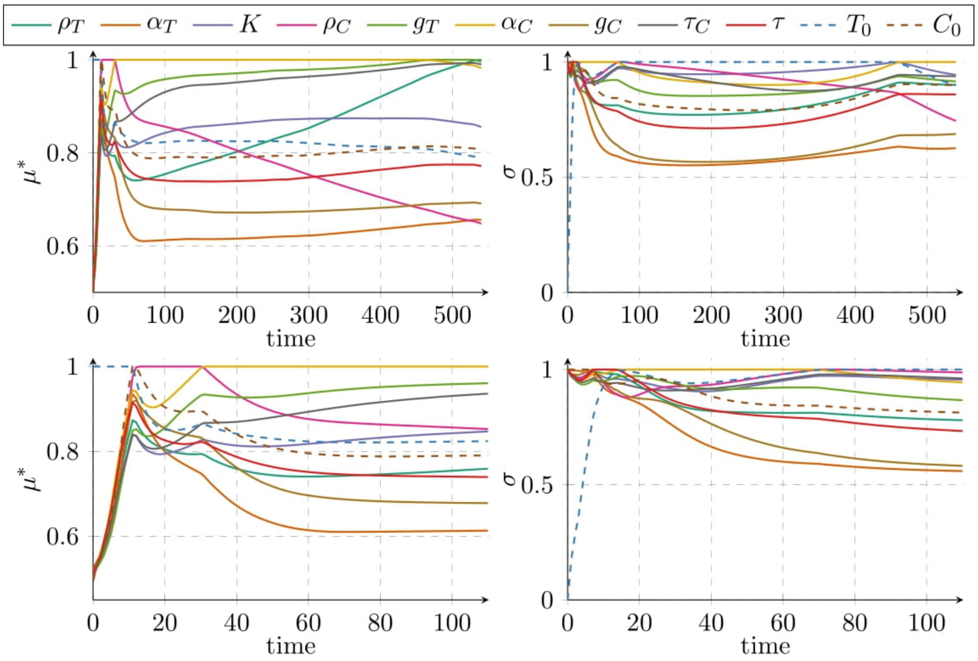

Figure S9: Sensitivity analysis results for the variable T (tumor cell population) under the **third approach** Migliorini et al. (2018), involving a single CAR-T dose modeled by Eqs. (S3) with initial conditions (S5) over the first 540 days of treatment. Colors represent parameters or initial conditions, as shown in the legend. Simulation parameters are listed in Table 1 (main text), with C_0 marked by ($\star\star$). The μ_i^* plots show the impact of specific model parameters on tumor cell population size, while the σ_i plots highlight parameters driving nonlinear and interactive effects in the model.

demonstrated a comparable and substantial influence, except for g_T and α_T, the latter continuing to have the least significance. By 540 days, the CAR-T proliferation rate ρ_C emerged as the most critical determinant of CAR-T cell population dynamics. Notably, ρ_T and C_0 became equally influential relative to the other parameters. Interestingly, the influence of α_T increased, while τ_C had the least impact at this final time point.

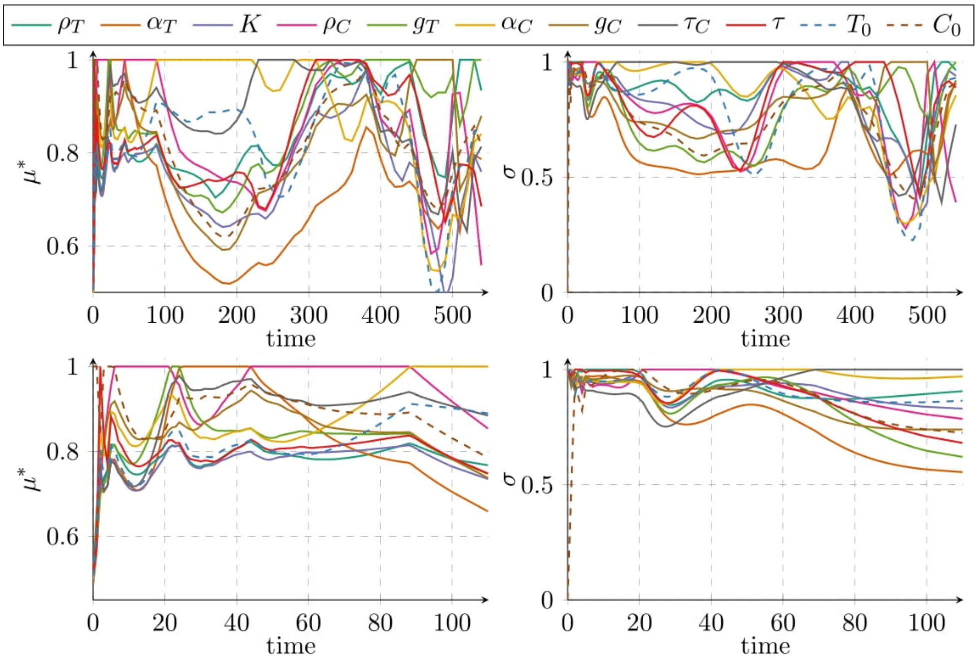

Figure S10: Sensitivity analysis results for the variable C (CAR-T cell population) under the **third approach** Migliorini et al. (2018), involving a single CAR-T dose modeled by Eqs. (S3) with initial conditions (S5) over the first 540 days of treatment. Colors represent parameters or initial conditions, as shown in the legend. Simulation parameters are listed in Table 1 (main text), with C_0 marked by ($\star\star$). The μ_i^* plots show the impact of specific model parameters on CAR-T cell population size, while the σ_i plots highlight parameters driving nonlinear and interactive effects in the model.

REFERENCES

Bodnar, M., Foryś, U., Piotrowska, M. J., Bodzioch, M., Romero-Rosales, J. A., and Belmonte-Beitia, J. (2023). On the analysis of a mathematical model of CAR-T cell therapy for glioblastoma: Insights from a mathematical model. *International Journal of Applied Mathematics and Computer Science* 33, 379–394. doi:10.34768/amcs-2023-002

Bodnar, M., Piotrowska, M. J., Bodzioch, M., Belmonte-Beitia, J., and Foryś, U. (2025). Dual CAR-T cell therapy for glioblastoma: strategies to cure tumour diseases based on a mathematical model. *Nonlinear Dynamics* 113, 1637–1666. doi:10.1007/s11071-024-10258-x

Migliorini, D., Dietrich, P.-Y., Stupp, R., Linette, G. P., Avery D. Posey, J., and June, C. H. (2018). CAR T-cell therapies in glioblastoma: A first look. *Clinical Cancer Research* 24, 535 – 540. doi:10.1158/1078-0432

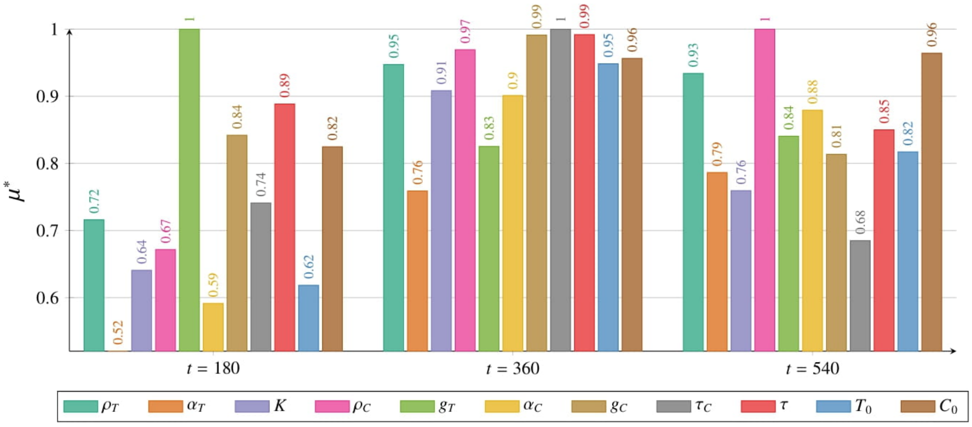

Figure S11: The impact of individual parameters and initial conditions on the CAR-T cell population under the **third approach** modeled by Eqs. (S3) with initial conditions (S5). Results are shown at 180 days (left), 360 days (middle), and 540 days (right). Colors represent parameters or initial conditions, as indicated in the legend. The vertical axis shows the μ_i^* measure. Simulation parameters are listed in Table 1 (in the main text), with C_0 marked as $(\star\star)$.

Qian, G. and Mahdi, A. (2020). Sensitivity analysis methods in the biomedical sciences. *Mathematical Biosciences* 323, 108306. doi:10.1016/j.mbs.2020.108306

Szafrańska-Łęczycka, M., Bodnar, M., Piotrowska, M. J., Krukowski, M., Belmonte-Beitia, J., and Foryś, U. (2025). Influence of time delay on the dynamics of a mathematical model of CAR-T cell therapy with logistic tumor growth. *Discrete and Continuous Dynamical Systems - B* 30, 4498–4515. doi:10.3934/dcdsb.2025073
